# HGPEC: a Cytoscape app for prediction of novel disease-gene and disease-disease associations and evidence collection based on a random walk on heterogeneous network

**DOI:** 10.1186/s12918-017-0437-x

**Published:** 2017-06-15

**Authors:** Duc-Hau Le, Van-Huy Pham

**Affiliations:** 1 Vinmec Research Institute of Stem Cell and Gene Technology, 458 Minh Khai, Hai Ba Trung, Hanoi, Vietnam; 2 Thuyloi University, 175 Tay Son, Dong Da, Hanoi, Vietnam; 3 Faculty of Information Technology, Ton Duc Thang University, Ho Chi Minh City, Vietnam

**Keywords:** Cytoscape app, Disease-gene association, Disease-disease association, Random walk with restart algorithm, Heterogeneous network, Gene prioritization, Disease prioritization

## Abstract

**Background:**

Finding gene-disease and disease-disease associations play important roles in the biomedical area and many prioritization methods have been proposed for this goal. Among them, approaches based on a heterogeneous network of genes and diseases are considered state-of-the-art ones, which achieve high prediction performance and can be used for diseases with/without known molecular basis.

**Results:**

Here, we developed a Cytoscape app, namely HGPEC, based on a random walk with restart algorithm on a heterogeneous network of genes and diseases. This app can prioritize candidate genes and diseases by employing a heterogeneous network consisting of a network of genes/proteins and a phenotypic disease similarity network. Based on the rankings, novel disease-gene and disease-disease associations can be identified. These associations can be supported with network- and rank-based visualization as well as evidences and annotations from biomedical data. A case study on prediction of novel breast cancer-associated genes and diseases shows the abilities of HGPEC. In addition, we showed prominence in the performance of HGPEC compared to other tools for prioritization of candidate disease genes.

**Conclusions:**

Taken together, our app is expected to effectively predict novel disease-gene and disease-disease associations and support network- and rank-based visualization as well as biomedical evidences for such the associations.

**Electronic supplementary material:**

The online version of this article (doi:10.1186/s12918-017-0437-x) contains supplementary material, which is available to authorized users.

## Background

The goal of gene and disease prioritization, one of the challenging issues in biomedicine, is to predict the most promising genes and diseases associated with a disease of interest. Many network-based methods have been proposed for this purpose [1, 2]. Among them, methods based on a heterogeneous network of genes and diseases are proven to outperform those solely based on a homogeneous network of genes/proteins [3–5]. In addition, these methods can not only prioritize candidate genes but also diseases; therefore, not only novel disease-gene relationships but also novel disease-disease associations can be identified. Moreover, prediction of novel genes associated with a disease, of which molecular basis is unknown, can be performed. In parallel with the proposed methods, a number of tools have been developed. However, they only focus on prediction of disease-gene associations [6, 7].

In a recent study, we have developed a tool, namely GPEC [8], which uses a random walk with restart (RWR) algorithm on a homogeneous network of genes/proteins to prioritize candidate genes. This RWR-based method is state-of-the-art among ones solely based on protein interaction network [9]. However, it can only prioritize candidate genes of diseases with known molecular basis and cannot directly figure out novel disease-disease associations.

Recently, a variant of RWR algorithm on a heterogeneous network, namely RWRH, has been proposed and used to identify novel disease-gene and disease-disease associations on a heterogeneous network of genes and diseases [3]. This method was proven to overcome limitations of the RWR-based method. More importantly, the RWRH algorithm can be extended to use any network of genes/proteins in the heterogeneous network. Indeed, a recent RWRH-based method has used a semantic similarity network of genes instead of the protein interaction network [10] and shown to outperform the original one [3]. We also note that there is no tool which employs this method available in public domain [11]. Therefore, we develop a tool, namely HGPEC, for identification of novel disease-gene and disease-disease associations. This tool can make use those advances of the RWRH-based method.

A common issue of gene prioritization tools is collection of biomedical evidence for novel promising associations between highly ranked genes and the disease of interest [6, 7]. For instance, network-based tools such as PRINCIPLE [12] and NetworkPrioritizer [13] just provide rankings for candidate genes but do not support evidences for associations between highly ranked genes and the disease of interest. In GPEC, we employed gene ontology [14], KEGG pathway [15], GeneRIF [16], PubMed [17], and OMIM [18] to support novel promising disease-gene associations. Note that, recent studies have demonstrated roles of shared known disease-associated genes, protein complexes, pathways and disease ontologies [19–22] in disease-disease associations. Therefore, in HGPEC, we additionally used protein complexes from CORUM [23] and terms from Disease Ontology [24] to support novel promising disease-disease associations.

To demonstrate functions of HGPEC, we first showed its ability in predicting novel genes and diseases associated with breast cancer. To this end, we selected top 20 ranked candidate genes and top 20 ranked candidate diseases, then performed visualization and evidence collection. Visualization results showed that most of the top ranked candidate genes are directly connected to known breast cancer-associated genes. Also, the top ranked candidate diseases are directly connected to either breast cancer or known breast cancer-associated genes. In addition to visualization, we collected evidences for promising associations between the top ranked candidate genes/diseases and breast cancer. Evidence collection results showed that each of the promising associations between the top ranked candidate genes and breast cancer is supported by at least two data sources. Meanwhile, seventeen out of the top 20 ranked candidate diseases have at least one gene, one pathway, one protein complex or one disease ontology term shared with breast cancer. Three remaining ones are highly phenotypically similar to breast cancer since they are directly connected to breast cancer in the phenotypic disease similarity network. Second, we compared the overall prediction performance of HGPEC with other tools, GPEC [8] and PRINCIPLE [12]. Simulation result on 330 diseases showed that HGPEC is much superior to these tools for prediction of disease-associated genes.

## Methods

Ranking/prioritization of candidate genes/diseases is to predict novel genes/diseases associated with a disease of interest. In this section, we first provide a summary of the RWRH-based method, which is used for ranking candidate genes/diseases in HGPEC. Then, we describe the implementation and databases used in HGPEC.

### RWRH-based method

**Fig. 1:**
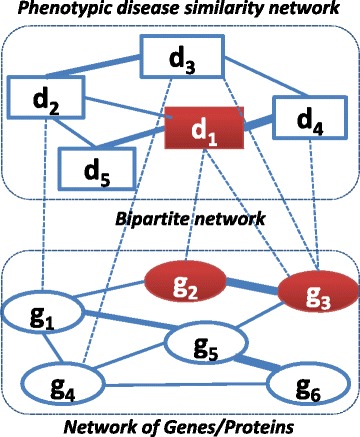
An illustrative heterogeneous network of genes and diseases. A phenotypic disease similarity network and a network of genes/proteins are connected by known disease-gene associations (i.e., a bipartite network) to form a heterogeneous network of genes and diseases

The heterogeneous network of genes and diseases can be represented as a connected weighted graph G(*V*, *E*) with a set of nodes *V* = {*v*_*1*_ *, v*_*2*_ *, …, v*_*N*_}, a set of links *E* = {(*v*_*i*_ *, v*_*j*_)| *v*_*i*_ *, v*_*j*_∈*V*} and a *N* × *N* adjacency matrix *W* of link weights. Figure 1 shows an illustrative heterogeneous network of genes and diseases. Given a disease of interest *d*_1_, the rankings of candidate genes/diseases are based on their relative importance to a set of source *S* ⊆ *V* (including *d*_1_ and known *d*_1_-associated genes). The relative importance measures how much a candidate gene/disease is associated with *d*_1_. Here, we introduce the RWRH algorithm for such task. This algorithm was proposed for prediction of disease-associated genes on a heterogeneous network of genes and diseases [3, 10, 25], drug-target interaction prediction on a heterogeneous network of drugs and targets [26] and identification of novel disease-microRNA associations based on heterogeneous network of diseases and microRNAs [27].

RWRH mimics a walker that moves from a current node to a randomly selected adjacent node or goes back to source nodes with a back-probability *γ*∈(0, 1) in a heterogeneous network. RWRH was formally defined as follows:

$$ {P}^{t+1}=\left(1-\gamma \right){W}^{\prime }{P}^t+{\gamma P}^0 $$

where *P* ^*t*^ is a *N* × 1 probability vector of all nodes in the network at a time step *t* of which the *i*th element represents the probability of the walker being at node *v*_*i*_∈*V*, and *P* ^0^ is the *N* × 1 initial probability vector. *W* ^′^is the transition matrix of the graph, the (*i*, *j*) element in *W* ^′^, denotes a probability with which a walker at *v*_*i*_ moves to *v*_*j*_ among *V*\{*v*_*i*_}. All nodes in the network are eventually ranked according to the steady-state probability vector *P* ^∞^. The steady-state of each node represents its relative importance to the set of source nodes *S*.

For the heterogeneous network of diseases and genes, the transition matrix *W* ^′^was defined as follows:

$$ {W}^{\prime }=\left[\begin{array}{cc}{W}_G^{\prime }& {W}_{G D}^{\prime}\\ {}{W}_{D G}^{\prime }& {W}_D^{\prime}\end{array}\right] $$

where${W}_G^{\prime }$ and ${W}_D^{\prime }$ are intra-subnetwork transition matrices of the network of genes/proteins and the phenotypic disease similarity network, respectively. ${W}_{GD}^{\prime }$, ${W}_{DG}^{\prime }$ are inter-subnetwork transition matrices. Let *λ* be the jumping probability the random walker jumps from the network of genes/proteins to the phenotypic disease similarity network or vice versa. Then, these matrices were defined as follows:

$$ {\left({W}_{GD}^{\prime}\right)}_{i, j}= p\left({d}_j|{g}_i\right)=\left\{\begin{array}{c}\frac{\uplambda {\left({W}_{GD}\right)}_{i j}}{\sum_j{\left({W}_{GD}\right)}_{i j}}\kern1.25em  if{\sum}_j{\left({W}_{GD}\right)}_{i j}\ne 0\\ {}0\kern9em  otherwise\end{array}\right. $$

$$ {\left({W}_{DG}^{\prime}\right)}_{i, j}= p\left({g}_j|{d}_i\right)=\left\{\begin{array}{c}\frac{\uplambda {\left({W}_{GD}\right)}_{j i}}{\sum_j{\left({W}_{GD}\right)}_{j i}}\kern1.5em  if{\sum}_j{\left({W}_{GD}\right)}_{j i}\ne 0\\ {}0\kern9.25em  otherwise\end{array}\right. $$

$$ {\left({W}_G^{\prime}\right)}_{i, j}=\left\{\begin{array}{c}\frac{{\left({W}_G\right)}_{i j}}{\sum_j{\left({W}_G\right)}_{i j}}\kern1.5em  if{\sum}_j{\left({W}_{G D}\right)}_{i j}=0\\ {}\frac{{\left(1-\uplambda \right)\left({W}_G\right)}_{i j}}{\sum_j{\left({W}_G\right)}_{i j}}\kern2.75em  otherwise\end{array}\right. $$

$$ {\left({W}_D^{\prime}\right)}_{i, j}=\left\{\begin{array}{c}\frac{{\left({W}_D\right)}_{i j}}{\sum_j{\left({W}_D\right)}_{i j}}\kern2.5em  if{\sum}_j{\left({W}_{GD}\right)}_{j i}=0\\ {}\frac{\left(1-\uplambda \right){\left({W}_D\right)}_{i j}\ }{\sum_j{\left({W}_D\right)}_{i j}}\kern3.5em  otherwise\end{array}\right. $$

where *W*_*G*_ , *W*_*D*_ and *W*_*GD*_ are adjacency matrices of the gene/protein, the phenotypic disease similarity and the bipartite networks, respectively.

By letting η be the parameter to weight the importance of each network, the initial probability vector was defined as follows:

$$ {P}^0=\left\{\begin{array}{c}\begin{array}{c}\left(1-\upeta \right)\frac{1}{\left| S\right|}\kern1.5em  if{v}_i\in S\\ {}\upeta \kern6em  if{v}_i\equiv {d}_1\end{array}\\ {}0\kern4.75em  otherwise\end{array}\right. $$

In case we are interested in a disease class/group, which contains set of diseases (*D*), we additionally define *P* ^0^ as follows:

$$ {P}^0=\left\{\begin{array}{c}\begin{array}{c}\left(1-\upeta \right)\frac{1}{\left| S\right|}\kern1.75em  if{v}_i\in S\\ {}\upeta \frac{1}{\left| D\right|}\kern4em  if{v}_i\in D\end{array}\\ {}0\kern4.75em  otherwise\end{array}\right. $$

All remaining diseases in the phenotypic disease similarity network are specified as candidate diseases, whereas candidate genes can be specified by users in different ways such as all remaining genes, neighbors of known associated genes, etc...

## Implementation

**Fig. 2:**
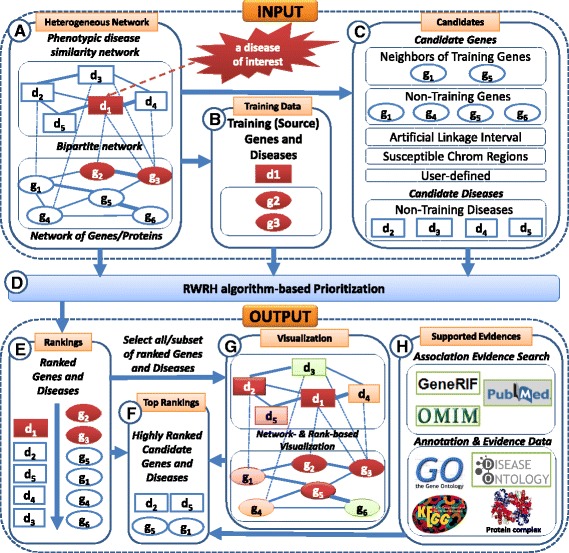
Implementation of HGPEC. Implementation of HGPEC consists of following steps: i) Constructing a heterogeneous network of genes and diseases (Part *A*); ii) Giving a disease of interest (*d*_1_) and identifying training (source) genes and diseases (Part *B*); iii) Providing candidate genes and diseases (Part *C*); iv) Prioritizing all genes and diseases in the network (Part *D*); and v) Displaying all ranked genes and diseases (Part *E*) and selecting highly ranked candidate genes and diseases for further investigation (Part *F*). Novel promising disease-gene and disease-disease associations can be supported with network- and rank-based visualization (Part *G*) as well as evidences and annotations from biomedical data (Part *H*)

HGPEC is developed based on the RWRH-based method as an app of Cytoscape v3.x, which is a platform for data integration, network analysis and visualization [28]. Therefore, it can work on any operating system such as Windows, Linux and Mac OS X, where Cytoscape is designed to work on. Figure 2 shows the implementation of HGPEC. In particular, HGPEC runs on a heterogeneous network consisting of a phenotypic disease similarity network, a network of genes/proteins and a bipartite network (Part A of Fig. 2). Given a disease of interest, training data including the given disease and its known associated genes is specified (Part B of Fig. 2). Candidate gene and disease sets are then provided. In which, candidate disease set includes non-training diseases. Meanwhile, candidate gene set can be easily constructed in several ways such as neighbors of training genes in the network, neighbors of training genes in the same chromosome, non-training genes in the network, susceptible chromosome regions/bands and freely defined by user (Part C of Fig. 2). The RWRH-based method then uses the training data to rank all candidate genes and diseases in the heterogeneous network (Part D of Fig. 2). Ranked genes and diseases are shown for further investigation (Part E of Fig. 2). For instance, highly ranked candidate genes and diseases (Part F of Fig. 2) can be further investigated by: i) network- and rank-based visualization (Part G of Fig. 2) and ii) supporting evidences including annotations for genes/diseases and evidences for novel promising disease-gene and disease-disease associations with preinstalled and automatically retrieved biomedical data (Part H of Fig. 2). Beside preinstalled data of gene and protein, gene ontology annotation and KEGG pathway like those in GPEC, we additionally preinstalled other biomedical data such as protein complex from CORUM [23] and disease ontology [24]. These data is used to annotate and support evidences for novel promising gene-disease and disease-disease associations. In addition, such associations can be further supported with evidence searched from GeneRIF, PubMed and OMIM. In HGPEC, the network of genes/proteins can be freely provided by users. By default, we loaded a human protein interaction network containing 10,486 genes and 50,791 interactions collected from ftp://ftp.ncbi.nlm.nih.gov/gene/GeneRIF/interactions.gz. This is a collection of human protein interactions from BIND [29], BioGRID [30] and HPRD [31]. Meanwhile, the phenotypic disease similarity network was collected from MimMiner [32] and the bipartite network are known disease-gene associations collected from either DisGeNET [33] or OMIM (http://ftp.ncbi.nlm.nih.gov/gene/DATA/mim2gene_medgen) [18]. These networks were also preinstalled in the app.

## Results and discussion

### Case study: Prediction of novel breast cancer-associated genes and diseases

**Fig. 3:**
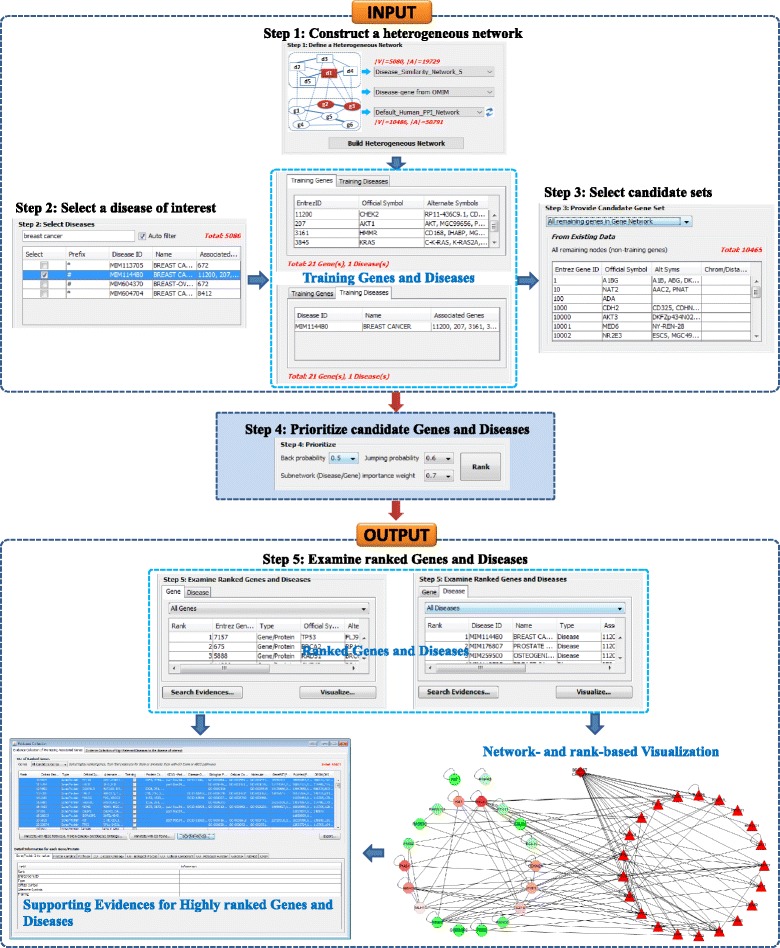
A workflow for prediction of novel breast cancer-associated genes and diseases. This task is completed after five following steps: *1*) Construct a heterogeneous network by selecting a phenotypic disease similarity network and a network of genes/proteins. *2*) Select breast cancer (OMIM ID: 114480, a disease of interest), then identify training genes (i.e., known breast cancer-associated genes) and training disease (i.e., breast cancer). *3*) Select a set of candidate genes; all remaining diseases in the network are selected as candidate diseases by default. *4*) Rank/prioritize all candidate genes and diseases by the RWRH-based method. *5*) Examine ranked genes and diseases by two means: ii) network- and rank-based visualization and ii) collection of annotations and association evidences for highly ranked candidate genes and diseases

Here, we show the ability of HGPEC in identifying novel genes and diseases associated with breast cancer (OMIM ID: 114480). Particularly, after ranking, sets of highly ranked candidate genes and diseases were further analyzed to find evidences about their associations with breast cancer. These associations were shown in a network-based view as well as evidences and annotations from biomedical data. To complete this task, we performed five following steps (see Fig. 3 and more detail in User manual in Additional file 3):

First, we constructed a heterogeneous network of genes and diseases. This network includes: i) a phenotypic disease similarity network containing 5080 diseases and 19,729 interactions, which was extracted from a phenotypic disease similarity matrix data collected from MimMiner [32] where only five interactions having largest weight to each disease were selected; ii) the default human protein interaction network and iii) the bipartite network containing known disease-gene associations collected from OMIM [18].

Second, we selected breast cancer (OMIM ID: 114480) for investigation. There are 21 known breast cancer-associated genes in the human protein interaction network. These genes and the disease of interest are played as training genes and disease.

Third, we selected all remaining genes (i.e., non-known breast cancer-associated genes) in the human protein interaction network as candidate genes and all remaining diseases in the phenotypic disease similarity network as candidate diseases. As a result, the candidate gene and disease sets include 10,465 genes and 5079 diseases, respectively.

Fourth, all genes and diseases in the heterogeneous network are ranked by applying the RWRH-based method with back-probability (i.e., *γ*), jumping probability (i.e., λ) and subnetwork importance weight (i.e., η) were set to 0.5, 0.6 and 0.7, respectively.

Finally, the associations between highly ranked candidate genes/diseases and breast cancer are then investigated by two means: i) Network- and rank-based visualization and ii) Collection of evidences including annotations for genes/diseases and evidences of promising associations.

**Fig. 4:**
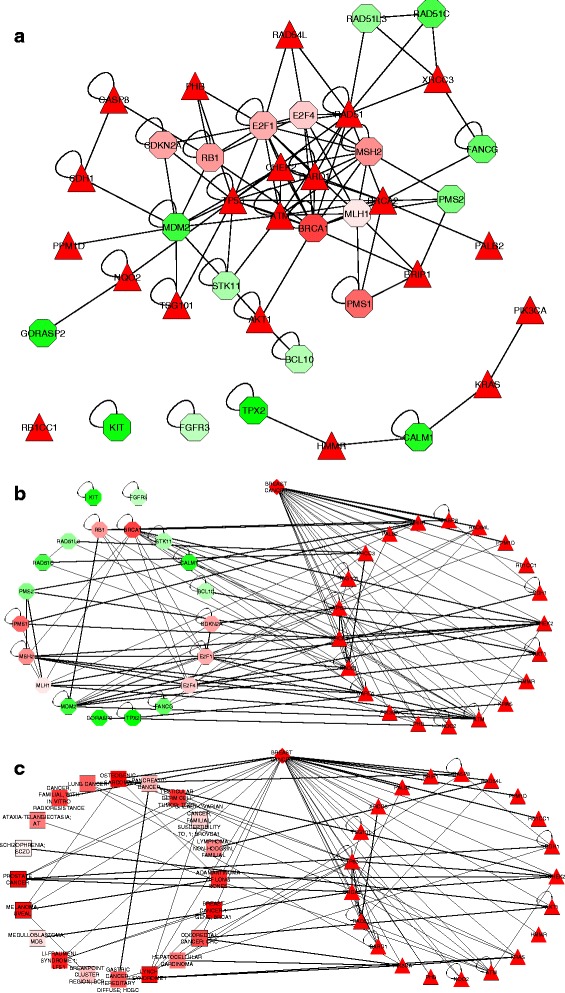
Topological relationships between highly ranked candidate genes/diseases and breast cancer. **a** Topological relationships between 20 highly ranked candidate genes and known breast cancer-associated genes in the human protein interaction network. **b** Topological relationships between 20 highly ranked candidate genes and breast cancer. **c** Topological relationship between 20 highly ranked candidate diseases and breast cancer. Nodes in octagon, rectangle, triangle and rhombus shape are candidate genes, candidate diseases, training genes and training disease, respectively. Nodes with high rankings are in *red*, relative high are in *pink*, medium are in *white* and *light green*, low are in *green*

For network- and rank-based visualization, we first investigated topological relationships between highly ranked candidate genes and breast cancer. To this end, we selected top 20 ranked candidate genes and 21 known breast cancer-associated genes for visualization. Fig. 4a and b show that most highly ranked genes are directly connected to known genes (only two candidate genes, *KIT* and *FGFR3*, are isolated). In addition, we explored topological relationships between highly ranked candidate diseases and breast cancer. More specifically, we selected top 20 ranked candidate diseases, breast cancer and its 21 known associated genes for visualization. Fig. 4c shows that highly ranked candidate diseases are directly connected to either breast cancer or the known breast cancer-associated genes. This means that candidate diseases which are phenotypically similar to or share known associated genes with the disease of interest are highly ranked.

For collection of evidences, we first collected annotations for highly ranked candidate genes and evidences for promising associations between them and breast cancer. In particular, we annotated the top 20 ranked genes with pathways, protein complexes, disease and gene ontology terms. Then, we collected evidences for promising associations between these genes and breast cancer from GeneRIF [16, 34], PubMed [35] and OMIM [18, 36]. As a result, at least one data source provides evidences for such the associations. In addition, all collected annotations and evidences for genes and promising disease-gene associations can be exported for further use (See Table S1 in Additional file 1). Second, we collected annotations and evidences for promising associations between highly ranked candidate diseases and breast cancer. To this end, we also annotated top 20 ranked candidate diseases with pathways, protein complexes, disease and gene ontology terms. Based on reports that common associated genes, protein complexes, pathways and annotated disease ontology terms can play important roles in disease-disease associations [19–22], we additionally checked whether or not these candidate diseases share genes, pathways, protein complexes and disease ontology terms with breast cancer. Table 1 shows that twelve of them (i.e., ranks: 1, 2, 6, 10, 11, 12, 13, 14, 15, 16, 19 and 20) have at least one gene, pathway, protein complex and disease ontology term shared with breast cancer. Meanwhile, five of them (i.e., ranks: 3, 4, 8, 17 and 18) have at least one pathway, protein complex and disease ontology term shared with breast cancer, but they do not share any gene with breast cancer. This means that if we only based on shared genes to associate these diseases with breast cancer, we could not find any association. However, other biomedical data such as pathway, protein complex and disease ontology can provide evidences for these associations. Finally, three remaining ones (Ranks: 5, 7 and 9) do not share any gene, pathway, protein complex or disease ontology term with breast cancer, but they have high rankings. This is because they are phenotypically similar to breast cancer as they are directly connected to it in the phenotypic disease similarity network (see Fig. 4c). Similarly, evidences of the promising associations between the selected candidate diseases and breast cancer can be collected from GeneRIF, PubMed and OMIM based on associations between their known associated genes and breast cancer. In addition, all collected annotations and evidences for diseases and promising disease-disease associations can be exported for further use (See Table S2 in Additional file 2). Moreover, all of the collected annotations and association evidences can be viewed in more detail in below panels (see User manual in Additional file 3).

**Table 1:** Evidence of associations between top 20 ranked diseases and breast cancer

Rank	OMIM ID	Name	# Shared genes	# Shared protein complexes	# Shared pathways	# Shared disease ontology
1	MIM176807	PROSTATE CANCER	3	10	19	71
2	MIM259500	OSTEOGENIC SARCOMA	2	14	23	55
3	MIM113705	BREAST CANCER 1 GENE; BRCA1	0	14	0	27
4	MIM120435	LYNCH SYNDROME I	0	1	2	35
5	MIM155720	MELANOMA, UVEAL	0	0	0	0
6	MIM151623	LI-FRAUMENI SYNDROME 1; LFS1	1	14	23	71
7	MIM102660	ADAMANTINOMA OF LONG BONES	0	0	0	0
8	MIM273300	TESTICULAR GERM CELL TUMOR; TGCT	0	3	8	34
9	MIM211410	CANCER, FAMILIAL, WITH IN VITRO RADIORESISTANCE	0	0	0	0
10	MIM114500	COLORECTAL CANCER; CRC	3	20	64	99
11	MIM137215	GASTRIC CANCER, HEREDITARY DIFFUSE; HDGC	3	8	60	93
12	MIM211980	LUNG CANCER	3	10	62	118
13	MIM114550	HEPATOCELLULAR CARCINOMA	3	27	62	81
14	MIM208900	ATAXIA-TELANGIECTASIA; AT	1	4	3	37
15	MIM605027	LYMPHOMA, NON-HODGKIN, FAMILIAL	1	1	3	5
16	MIM260350	PANCREATIC CANCER	2	14	45	54
17	MIM151410	BREAKPOINT CLUSTER REGION; BCR	0	0	1	10
18	MIM604370	BREAST-OVARIAN CANCER, FAMILIAL, SUSCEPTIBILITY TO, 1; BROVCA1	0	14	0	27
19	MIM155255	MEDULLOBLASTOMA; MDB	1	3	3	36
20	MIM181500	SCHIZOPHRENIA; SCZD	1	3	40	84

### Comparison to other network-based tools for prioritization of candidate disease gene

**Fig. 5:**
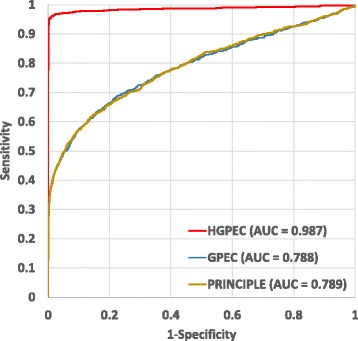
Performance comparison

Many web-based tools, which are based on different computational methods, have been introduced for disease gene prediction [6, 7]. These tools only focus on prioritization of candidate genes. In addition, some tools require users uploading their own data. Recently, a number of Cytoscape apps have been designed for disease gene prioritization. The underlying methods of these tools are network-based since they can utilize functions of network integration, analysis and visualization of Cytoscape. Indeed, PRINCIPLE [12] is a tool for associating genes with diseases via network propagation algorithm PRINCE [37]. Given a query disease, PRINCIPLE prioritizes candidate disease genes based on their closeness in a protein interaction network to genes causing phenotypically similar disorders to the query disease. Therefore, this tool cannot directly figure out novel disease-disease associations. In addition, novel disease-gene associations predicted by this tool are not provided with biomedical evidences. Another Cytoscape app, NetworkPrioritizer [13], which is also designed for prioritization of candidate disease genes. This tool computes a number of important centrality measures to rank nodes based on their relevance for network connectivity and provides different methods to aggregate and compare rankings. Based on the final rankings, novel disease-associated genes can be predicted. However, it has the same limitation as in PRINCIPLE because there is no function in NetworkPrioritizer which helps user to search evidences for predicted disease-gene associations. In addition, it is not designed to find novel disease-disease associations. As aforementioned, we have recently developed a Cytoscape app, GPEC [8], for disease gene prediction and evidence collection based on the RWR-based algorithm. This app was shown more useful than the above Cytoscape apps since it has functions for collecting biomedical evidences for predicted disease-gene associations. However, it also cannot directly predict novel disease-disease associations. In addition, like the above tools, it can only work with diseases with known molecular basis. Therefore, HGPEC is introduced to overcome all of these limitations. In addition, HGPEC is designed based on a state-of-the-art network-based method (i.e., RWRH-based method), which was shown to outperform the methods used in GPEC as well as PRINCIPLE. To compare overall performance of HGPEC with that of GPEC and PRINCIPLE, we used the human protein interaction network and set the best settings for the three methods as reported in previous studies [3, 37, 38] (i.e., back-probability and weight parameter were set to 0.5 in GPEC and PRINCIPLE, respectively. Meanwhile, back-probability, jumping probability and subnetwork importance weight were set to 0.5, 0.6 and 0.7 for HGPEC, respectively). Due to using leave-one-out cross validation method, we selected a set of 330 diseases with at least two known associated genes to compare the performance of these tools in terms of AUC (i.e., area under the ROC curve) values. Figure 5 shows that HGPEC (AUC = 0.987) performs much better than GPEC (AUC = 0.788) and PRINCIPLE (AUC = 0.789).

## Conclusions

HGPEC employs the random walk with restart algorithm in a heterogeneous network of genes and diseases. It is developed to overcome the limitations of existing disease gene prediction tools. Beside the capability of prioritization of candidate genes, HGPEC can also rank candidate diseases. Therefore, it can discover not only novel gene-disease associations but also new disease-disease associations. In addition, it can identify novel genes associated with diseases without known molecular basis. Moreover, it is also convenient for users with freedom input of network of genes/proteins. Furthermore, novel promising gene-disease and disease-disease associations can be supported with network- and rank-based visualization as well as evidences and annotations collected from biomedical data. A case study on prediction of novel breast cancer-associated genes and diseases was performed to show the abilities of HGPEC. In addition, we also showed that HGPEC is much better than other tools (i.e., GPEC and PRINCIPLE) in prioritizing candidate disease genes. Note that, disease similarity network (i.e., diseasome) can be constructed based on shared disease gene [19], shared pathways [21], shared miRNA [39], shared protein complex [40], shared disease ontology [22] and disease comorbidity [41]. Therefore, in our future study, the phenotypic disease similarity network will be replaced by any diseasome, which are able to be provided freely by users. Moreover, we are going constantly to upgrade HGPEC so that it will be compatible with latest Cytoscape series and therefore become more popular.

## Availability and requirements

• **Project name:** HGPEC

• **Project home page:** https://sites.google.com/site/duchaule2011/bioinformatics-tools/hgpec

• **Operating system(s):** Windows/Linux/MacOS

• **Programming language:** Java

• ** Other requirements:** Java 1.7 or higher, Cytoscape 3.x (Cytoscape 3.3 or higher)

• **License:** None

• **Any restriction to use by non-academics:** None

## Additional files

Additional file 1: Table S1. All collected annotations and evidences for associations between top 20 ranked candidate genes and breast cancer. (TXT 622 kb)

Additional file 2: Table S2. All collected annotations and evidences for associations between top 20 ranked candidate diseases and breast cancer. (TXT 278 kb)

Additional file 3: User manual. (PDF 2710 kb)
