# Supplementary material for: HGPEC: a Cytoscape app for prediction of novel disease-gene and disease-disease associations and evidence collection based on a random walk on heterogeneous network
Source: BMC Syst Biol. 2017 Jun 15;11:61. doi: 10.1186/s12918-017-0437-x (PMC5472867; doi:10.1186/s12918-017-0437-x)
Supplement: Supplementary file 3 — User manual. (PDF 2710 kb) [file 12918_2017_437_MOESM3_ESM.pdf]

Systems biology

HGPEC: a Cytoscape app for prediction of novel disease-gene and disease-disease associations and evidence collection based on a random walk on heterogeneous network

Duc-Hau Le[1,*]

[1]Vinmec Research Institute of Stem Cell and Gene Technology, 458 Minh Khai, Hai Ba Trung, Hanoi, Vietnam.

[*]To whom correspondence should be addressed.

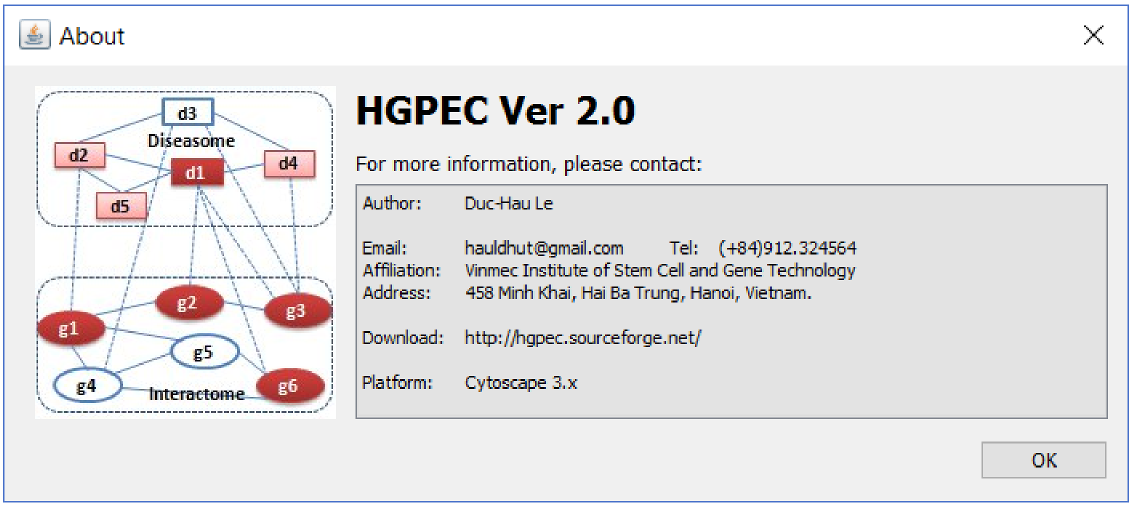

User Manual

I. Setup 2

II. Overview of HGPEC 3

III. Case study: Prediction of novel breast cancer-associated genes and diseases 4

III.1. Step 1: Construct a heterogeneous network 4

III.2. Step 2: Select a disease of interest 4

III.3. Step 3: Select candidate sets 5

III.4. Step 4: Prioritize candidate genes and diseases in the heterogeneous network 5

III.5. Step 5: Examine ranked genes and diseases 5

III.5.1. Visualization 5

Topological relationships between highly ranked candidate genes and the disease of interest 6

Topological relationships between highly ranked candidate diseases and the disease of interest 8

III.5.2. Annotation & Evidence Search 9

Annotation and evidence collection for associations between highly ranked candidate genes and the disease of interest 10

Annotation and evidence collection for relevance between highly ranked candidate diseases and the disease of interest 13

IV. Reference 16

I. Setup

- HGPEC 2.0 can only run on **Cytoscape 3.x** platform, therefore user should download Cytoscape version 3.x at http://cytoscape.org/
- Cytoscape need JRE to run, therefore download JRE version 7.x or later from http://www.oracle.com/technetwork/java/index.html and install it.
- Install Cytoscape to the root folder (e.g., C:\Program Files\Cytoscape_v3.4.0).
- Download HGPEC_v2.0.jar file from http://hgpec.sourceforge.net/ or https://sites.google.com/site/duchaule2011/bioinformatics-tools/hgpec. Then, install it by going to **Apps** → **App Manager**…. After that, choose **Install from file…**, then select the downloaded HGPEC_v2.0.jar file.

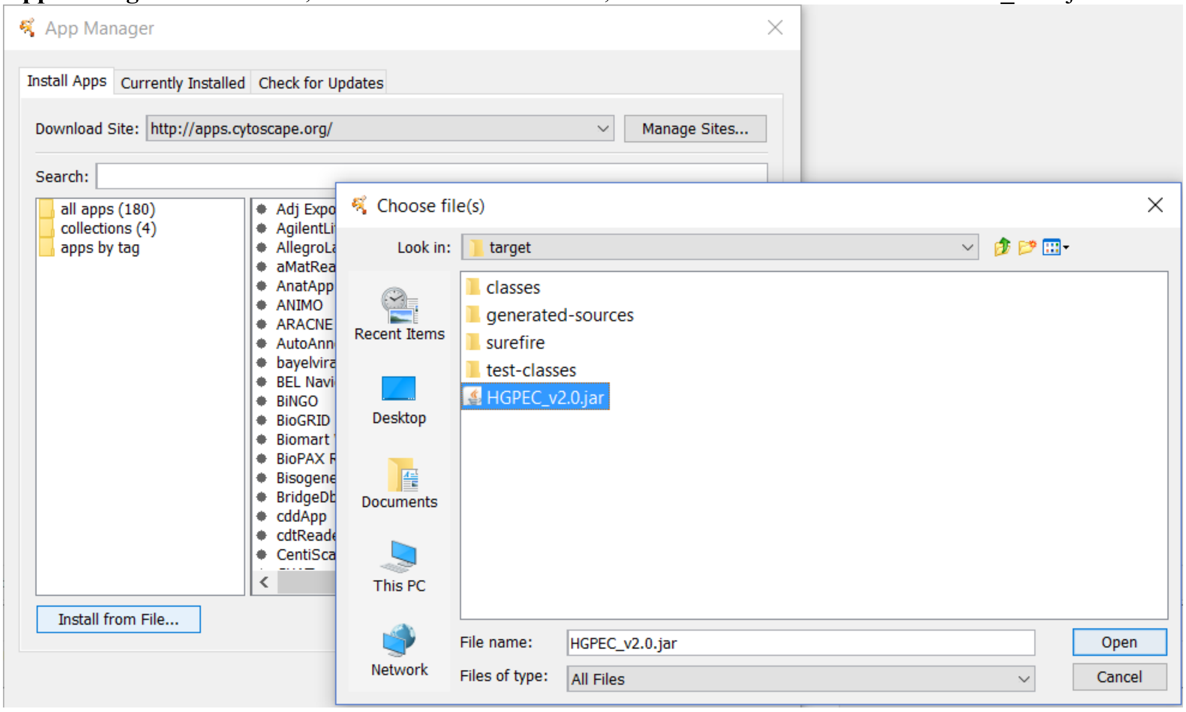

- Create folders **Data** in the root folder of Cytoscape (e.g., C:\Program Files\Cytoscape_v3.4.0).
- Download GO annotation and GeneRIF data at ftp.ncbi.nlm.nih.gov/gene/DATA/gene2go.gz and ftp://ftp.ncbi.nih.gov/gene/GeneRIF/generifs_basic.gz, respectively, then extract and store in the **Data** folder (e.g., C:\Program Files\Cytoscape_v3.4.0\Data).

- Note that: HGPEC_v2.0 can work on Windows, Ubuntu and Mac OS.

II. Overview of HGPEC

After installing, HGPEC will be automatically loaded in the App menu of Cytoscape

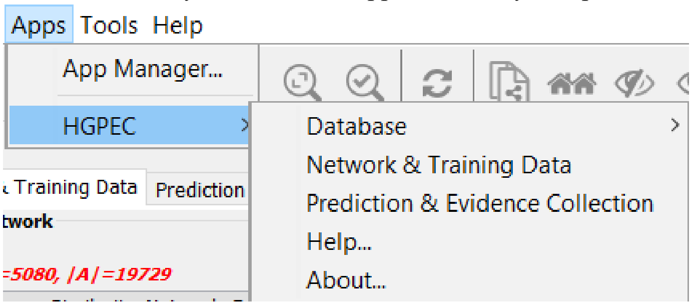

HGPEC has the following menu items:

- **Databases**: This shows pre-installed data in HGPEC. It also contains links to GO annotation and GeneRIF data sources.
- **Network & Training Data** and **Prediction & Evidence Collection**: These contain main functions of HGPEC corresponding to two following panels
- **Help…** and **About…**: To show user manual and additional information of HGPEC.

HGPEC is organized into two main panels (**Network & Training Data** and **Prediction & Evidence Collection**).

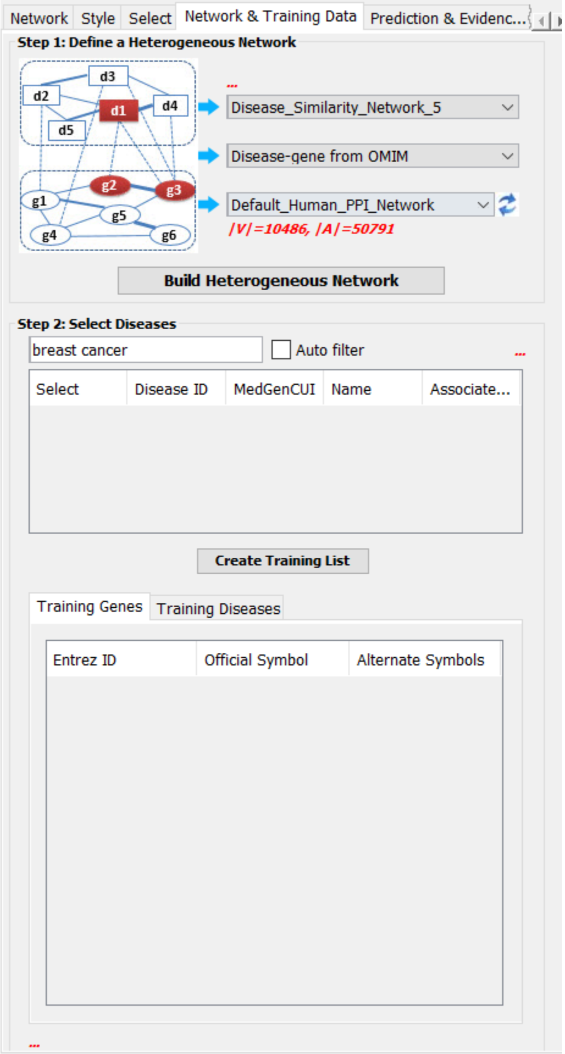

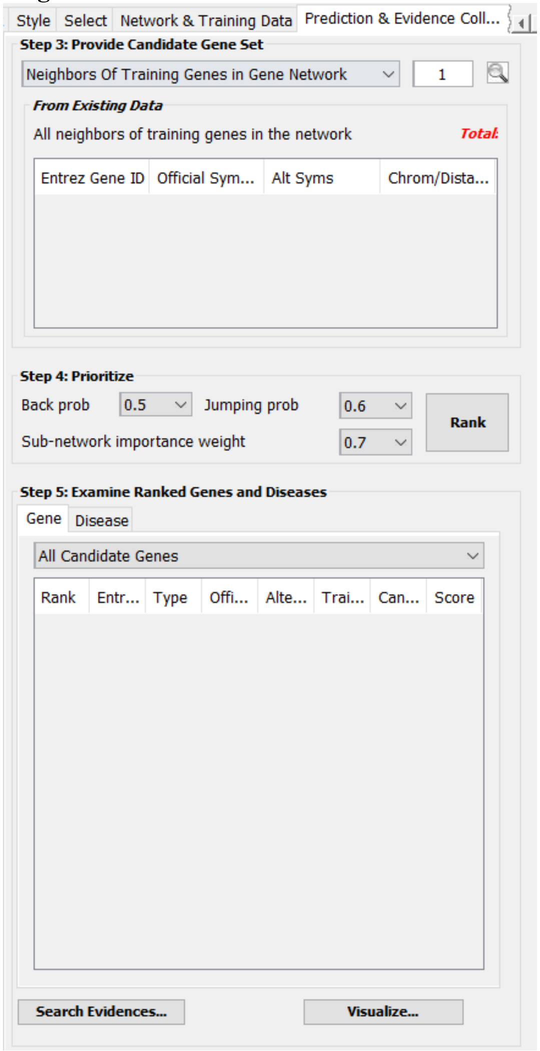

The main tasks (Prediction of Genes and Diseases, and Evidence Collection) of HGPEC are completed after five steps:

- **Step 1**: Define a Heterogeneous network
- **Step 2**: Select Diseases of interest
- **Step 3**: Provide Candidate Gene Set
- **Step 4**: Prioritize (candidate genes and diseases)
- **Step 5**: Examine Ranked Genes and Diseases

III. Case study: Prediction of novel breast cancer-associated genes and diseases

In the following section, we show the ability of HPEC in identifying novel breast cancer-associated genes and diseases.

III.1. Step 1: Construct a heterogeneous network

To this end, we select a phenotypic disease similarity network containing 5,080 diseases and 19,729 interactions (i.e., **Disease_Similarity_Network_5**) and a human protein interaction network containing 10,486 genes and 50,791 interactions (i.e., **Default_Human_PPI_Network**). Then, we connect them by known disease-gene associations from either OMIM (Amberger, et al., 2009) or DisGeNET (Piñero, et al., 2017) to construct a heterogeneous network of diseases and genes by clicking **Build Heterogeneous Network**

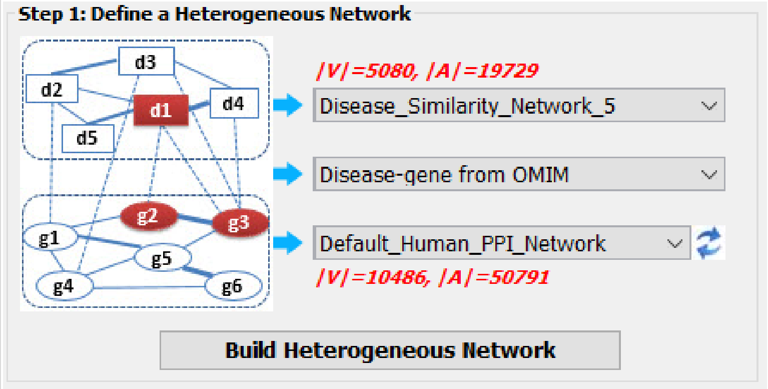

To construct a heterogeneous network:

1. *Select a disease similarity network.*
2. *Select known disease-gene associations*
3. *Select a network of genes/proteins (e.g., the preinstalled one or one imported from Cytoscape).*
4. *Click* **Build Heterogeneous Network** *to connect these two networks by the known disease-gene associations.*

Note that:

- *For disease similarity network: We pre-installed 3 networks corresponding to 5, 10 or 15 nearest neighbors, which were extracted from a phenotypic disease similarity matrix data collected from (van Driel, et al., 2006)*
- *For gene/protein interaction network:*
    - *We pre-installed a human physical protein interaction network collected from ftp://ftp.ncbi.nlm.nih.gov/gene/GeneRIF/interactions.gz.*
    - *However, user can use other protein/gene interaction networks by importing them to Cytoscape (File → Import → Network from table (Text/MS Excel)…). Genes/Proteins in the network must be identified by Gene Entrez ID. After that, click* 🔄 *to load the imported network to network list.*
- *For known disease-gene associations: User can select from either OMIM or DisGeNET*

III.2. Step 2: Select a disease of interest

We select breast cancer (OMIM ID: 114480), then create training list by click **Create Training List**.

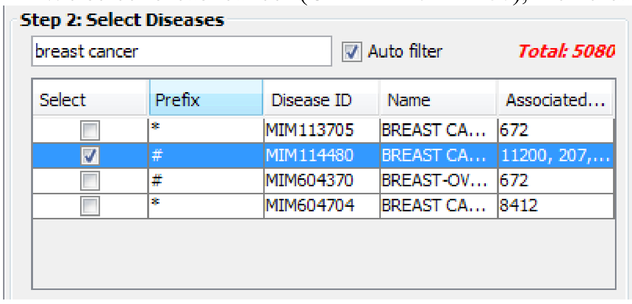

Note that:

- *To quickly select a disease of interest, user should type a keyword then check* **Auto filter**

As a result, the training list includes the disease of interest (OMIM ID: 114480) and its 21 known associated genes.

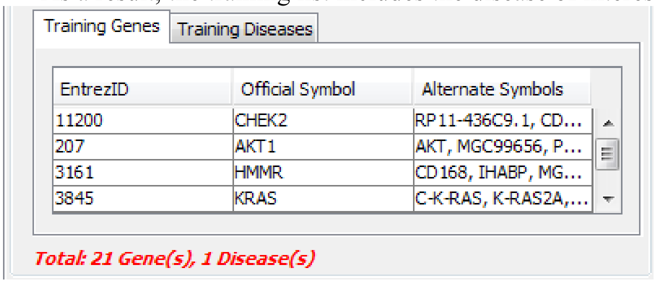

A total of 21 known associated genes

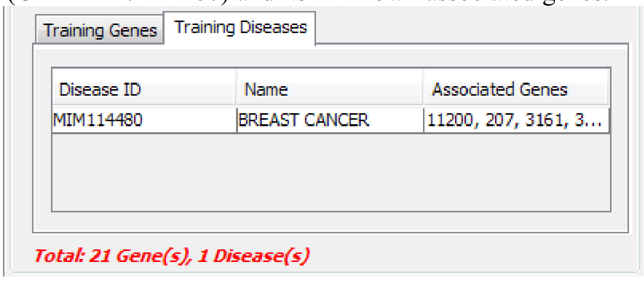

The disease of interest (OMIM ID: 114480)

III.3. Step 3: Select candidate sets

For candidate diseases, all remaining diseases are specified as candidate diseases by default. Therefore, there are 5,079 diseases in this set.

For candidate genes, we select option **All remaining genes in Gene Network**. As a result, a total of 10,465 remaining genes were selected as candidate genes.

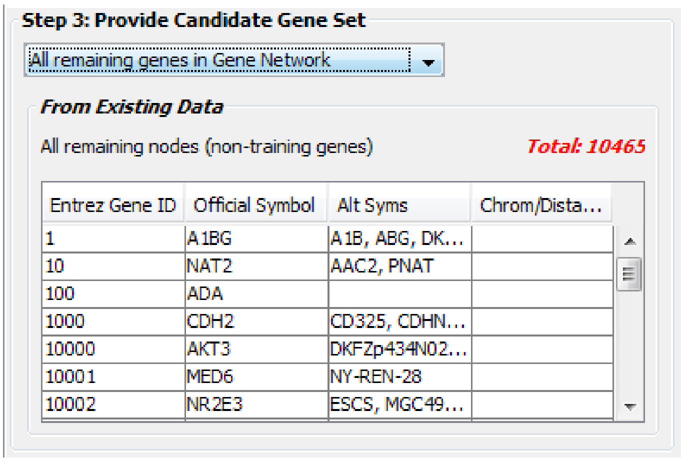

Five ways to construct a candidate gene set:

- **Neighbors of Training Genes in Gene Network**
  - User must define distance of neighbors to training genes
- **Neighbors Of Training Genes in Chromosome** (also known as **Artificial Linkage Interval**)
  - User must define number of neighbors of each training gene in the same chromosome.
- **All remaining genes in Gene Network**
- **Susceptible Chromosome Regions/Bands**
  - User selects candidate genes from susceptible chromosome regions/bands.
- **User-defined**
  - User manually provides a set of candidate genes by Entrez Gene ID or Gene Symbol.

III.4. Step 4: Prioritize candidate genes and diseases in the heterogeneous network

We set three parameters (i.e., back-probability (γ), jumping probability (λ) and subnetwork (Disease/Gene) importance (η)) of RWRH algorithm to 0.5, 0.6 and 0.7, respectively. Please refer to (Li and Patra, 2010) for best parameter setting.

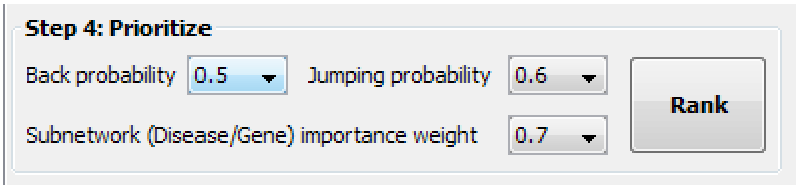

Then click 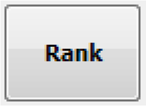 to rank all candidate genes and diseases in the heterogeneous network

III.5. Step 5: Examine ranked genes and diseases

Ranked genes and diseases are shown in two data tables

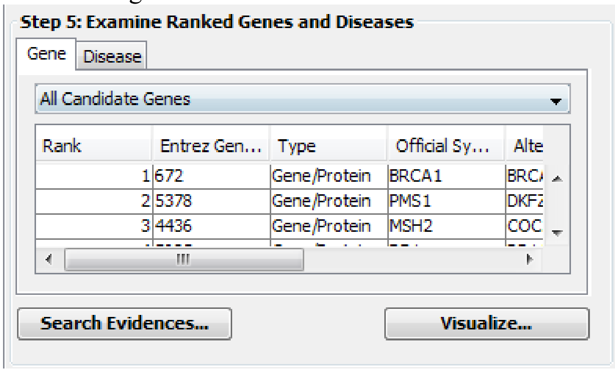

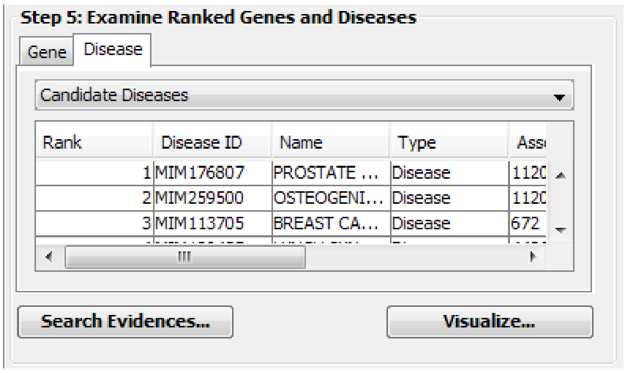

From this interface, user can visualize, annotate and search evidences for novel promising associations between highly ranked candidate genes/ diseases and the disease of interest.

III.5.1. Visualization

Note that, not only candidate genes and diseases are ranked, but all genes and diseases in the heterogeneous network are also ranked. Therefore, user can visualize them in one view to exploit their topologically relationships.

HGPEC provides two options of visualization (**Gene/Protein interaction network** and **Heterogeneous network**)

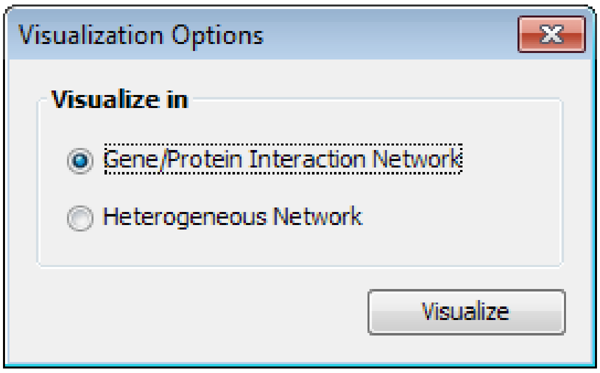

- Option **Gene/Protein interaction network**: Only relationships among selected genes in the gene/protein interaction network are visualized.
- Option **Heterogeneous network**: Relationships between selected genes and diseases in the heterogeneous network are visualized.

<u>Topological relationships between highly ranked candidate genes and the disease of interest</u>

- If we only focus on topological relationships between highly ranked candidate genes and known genes of disease of interest, we should select option **Gene/Protein interaction network**. For example, we selected 20 highly ranked candidate genes and 21 training genes of breast cancer for visualization

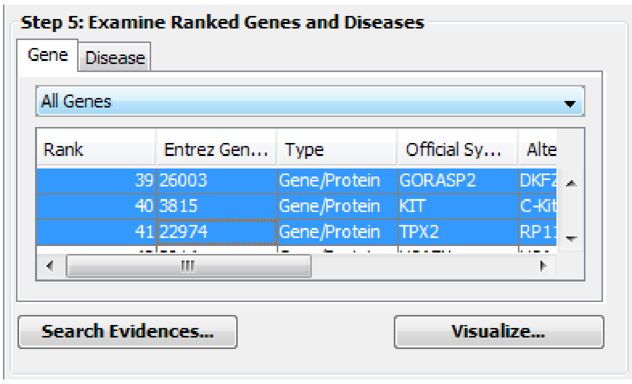

→

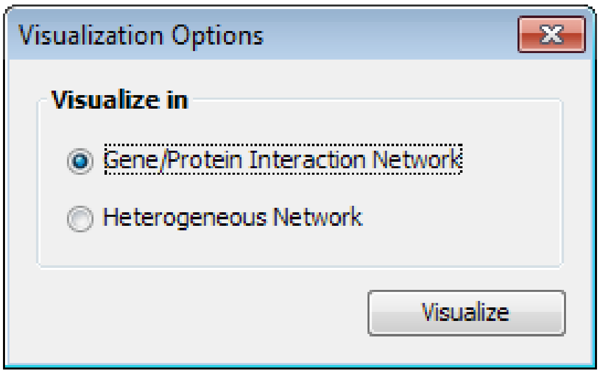

1. Select 21 training and 20 highly ranked candidate genes. Then click 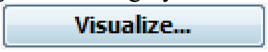

2. Select option **Gene/Protein interaction network**. Then click 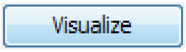

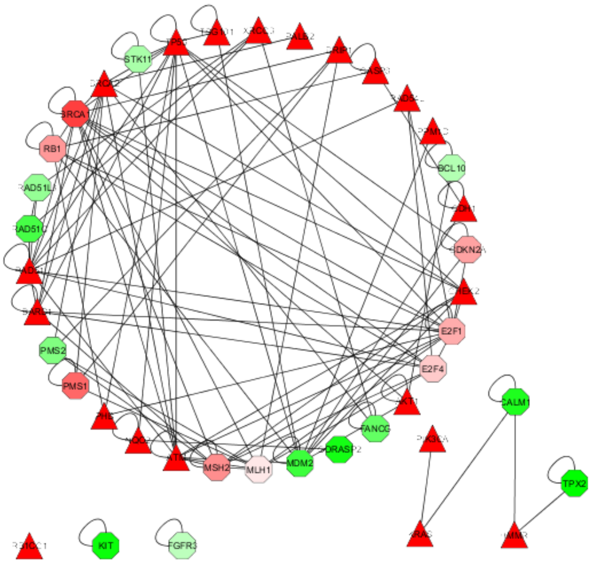

→

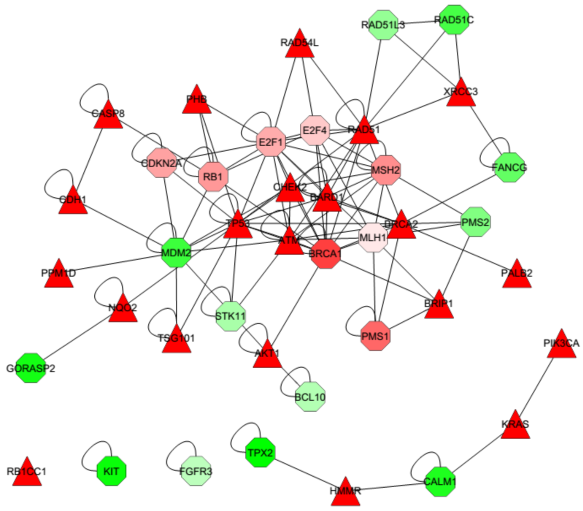

3. Group Attributes layout (default mode of HGPEC)

4. Switch to Force-directed Layout (Layout → Cytoscape Layouts → Force-directed Layout)

Nodes in triangle shape are training genes, remaining ones are candidates. Nodes with high rankings are in red, relative high are in pink, medium are in white and light green, low are in green.

We found that the sub-network are mostly connected. In other words, highly ranked genes are directly connected to known/training genes

- If we focus on topological relationships between highly ranked candidate genes and disease of interest and its associated genes, we should select option **Heterogeneous network**. For example, we selected top 20 ranked candidate genes, 21 training genes as above and the training disease (i.e., OMIM ID: 114480) for visualization

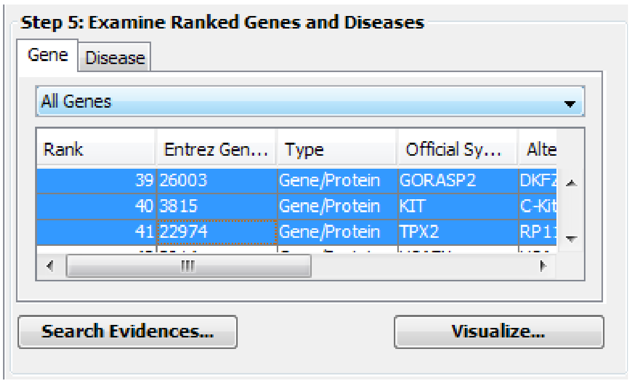

→

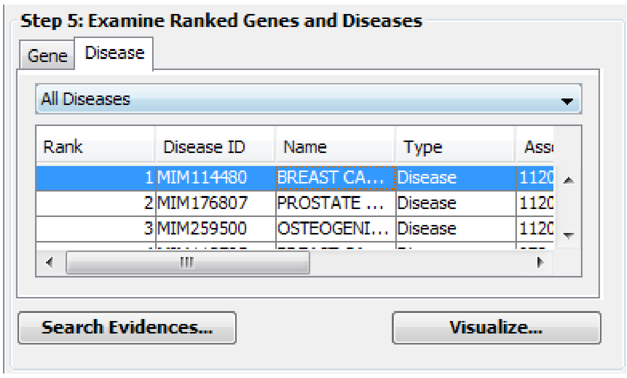

1. Select top 20 ranked candidate genes and 21 training genes.

2. Select the training disease (i.e., the disease of interest, OMIM ID: 114480)

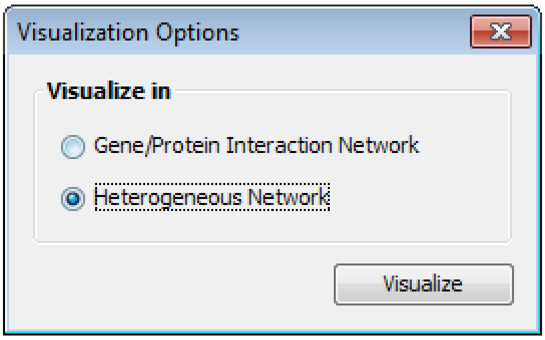

→

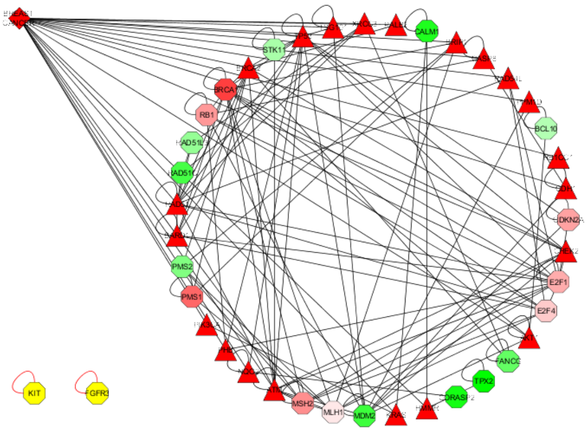

3. Select option **Heterogeneous interaction network**. Then click 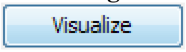

4. Group Attributes Layout with "Type" attribute (default mode of HGPEC)

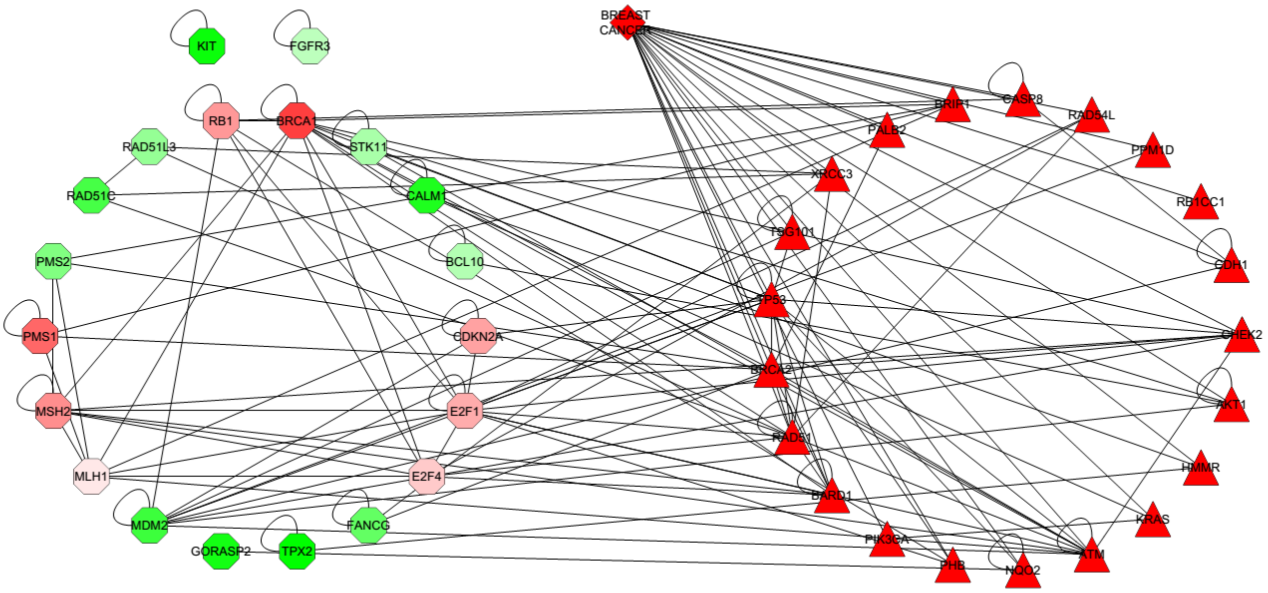

5. Switch to Group attribute layout with "Role" attribute (Layout → Cytoscape Layouts → Group Attributes Layout → Role)
Node in rhombus shape is the disease of interest. Nodes with high rankings are in red, relative high are in pink, medium are in white and light green, low are in green.

Topological relationships between highly ranked candidate diseases and the disease of interest

In this case, we selected top 20 ranked candidate diseases, 21 training genes and the disease of interest (i.e., OMIM ID: 114480) for visualization.

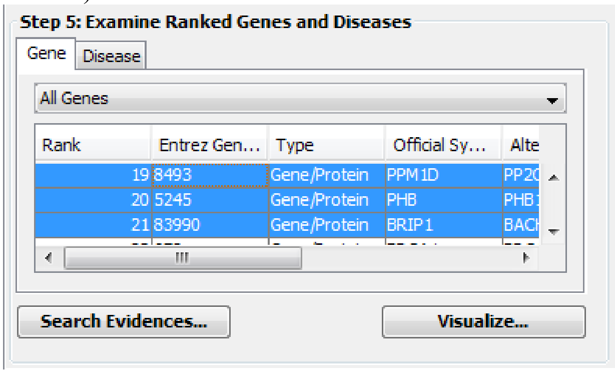

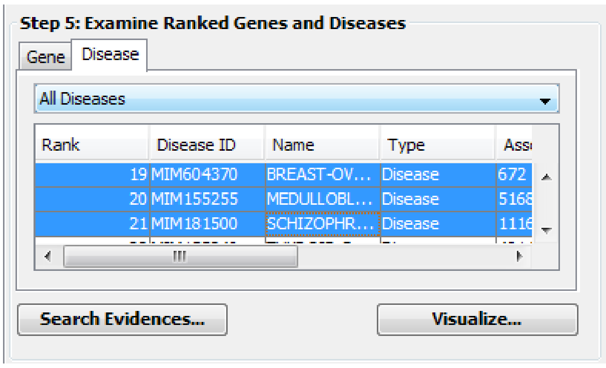

1. Select 21 training genes.

2. Select top 20 ranked candidate diseases and the training disease.

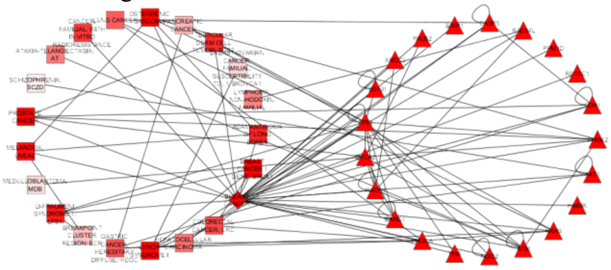

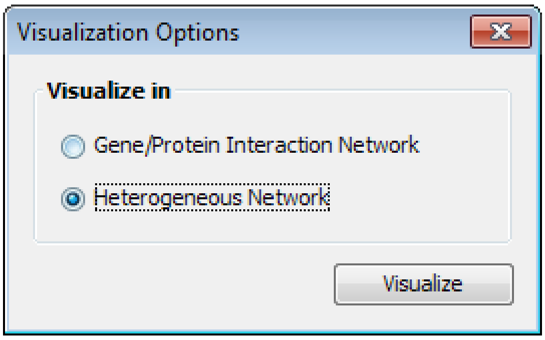

3. Select option **Heterogeneous interaction network**.
   Then click 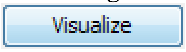

4. Group Attributes Layout with "Type" attribute (default mode of HGPEC)

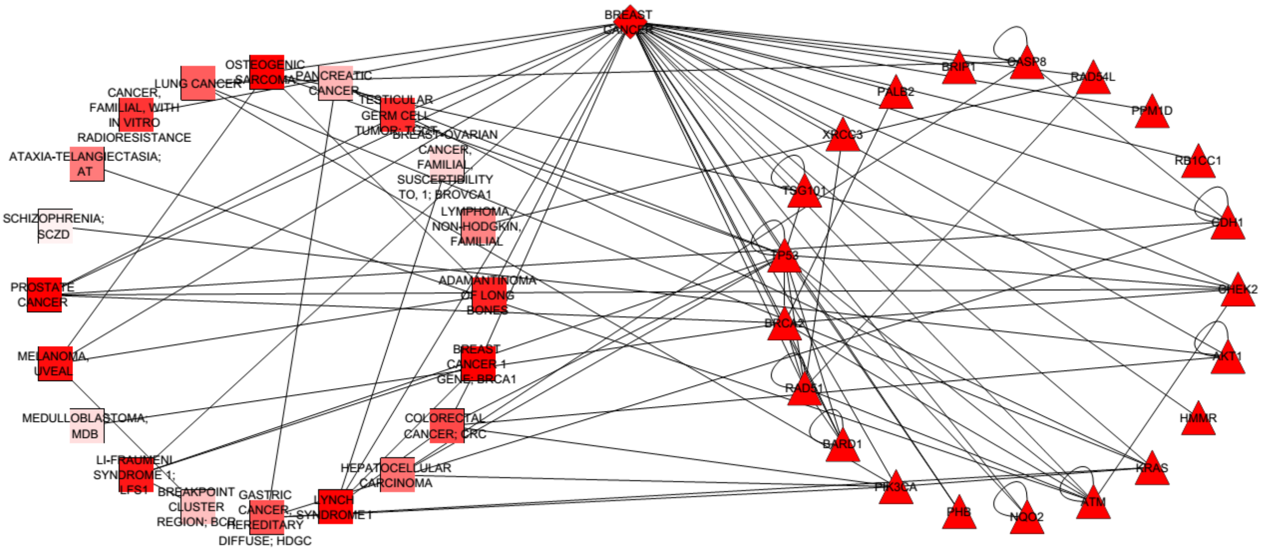

5. Switch to Group attribute layout with "Role" attribute (Layout → Cytoscape Layouts → Group Attributes Layout → Role)
   Node in rhombus shape is the disease of interest. Nodes in rectangle shape are candidate diseases. Nodes with high rankings are in red, relative high are in pink, medium are in white and light green, low are in green.

Similarly, we found that the sub-network are connected. In other words, highly ranked candidate diseases are directly connected to either known/training genes or the disease of interest. This means that candidate diseases which have connections to the disease of interest or associated with training genes are highly ranked.

III.5.2. Annotation & Evidence Search

This function is to collect evidences and annotations for associations between highly ranked candidate genes/diseases and the disease of interest.

Ranked genes and diseases are organized in two tabs

<u>Ranked genes</u>

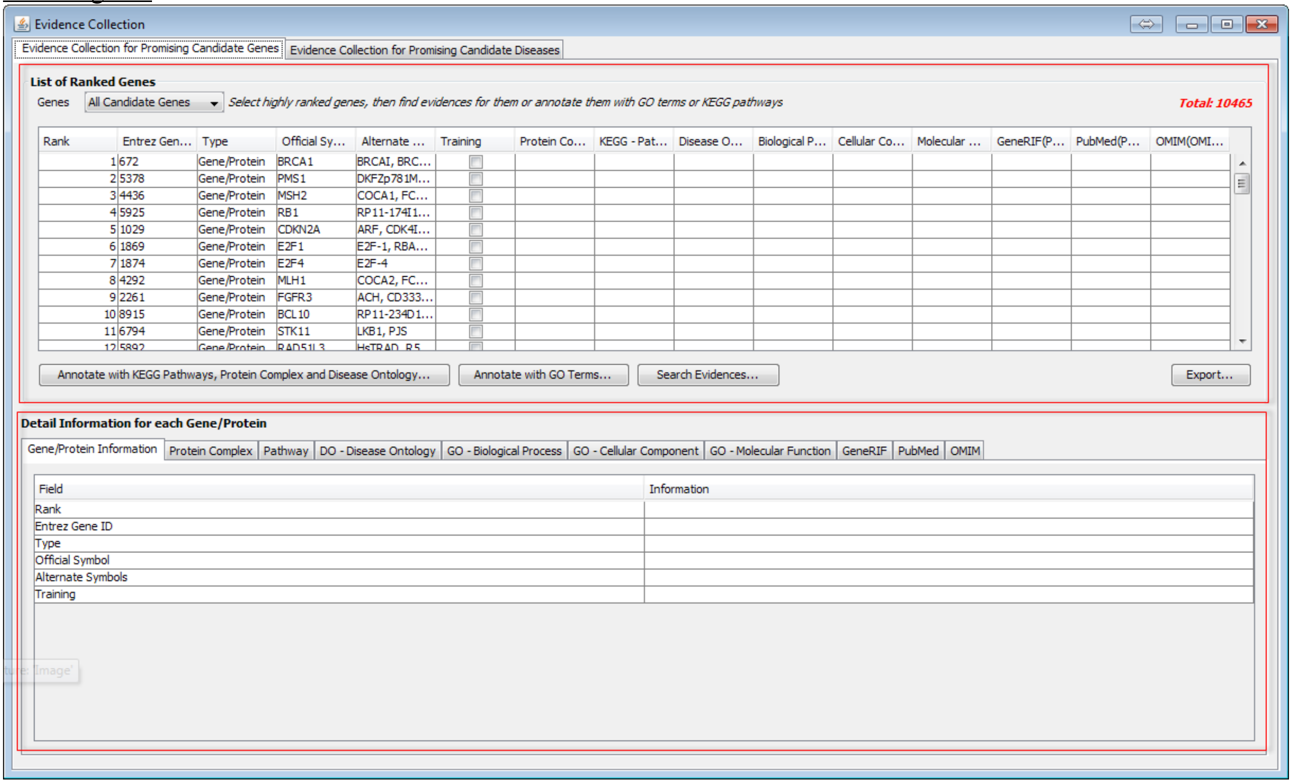

<u>Ranked diseases</u>

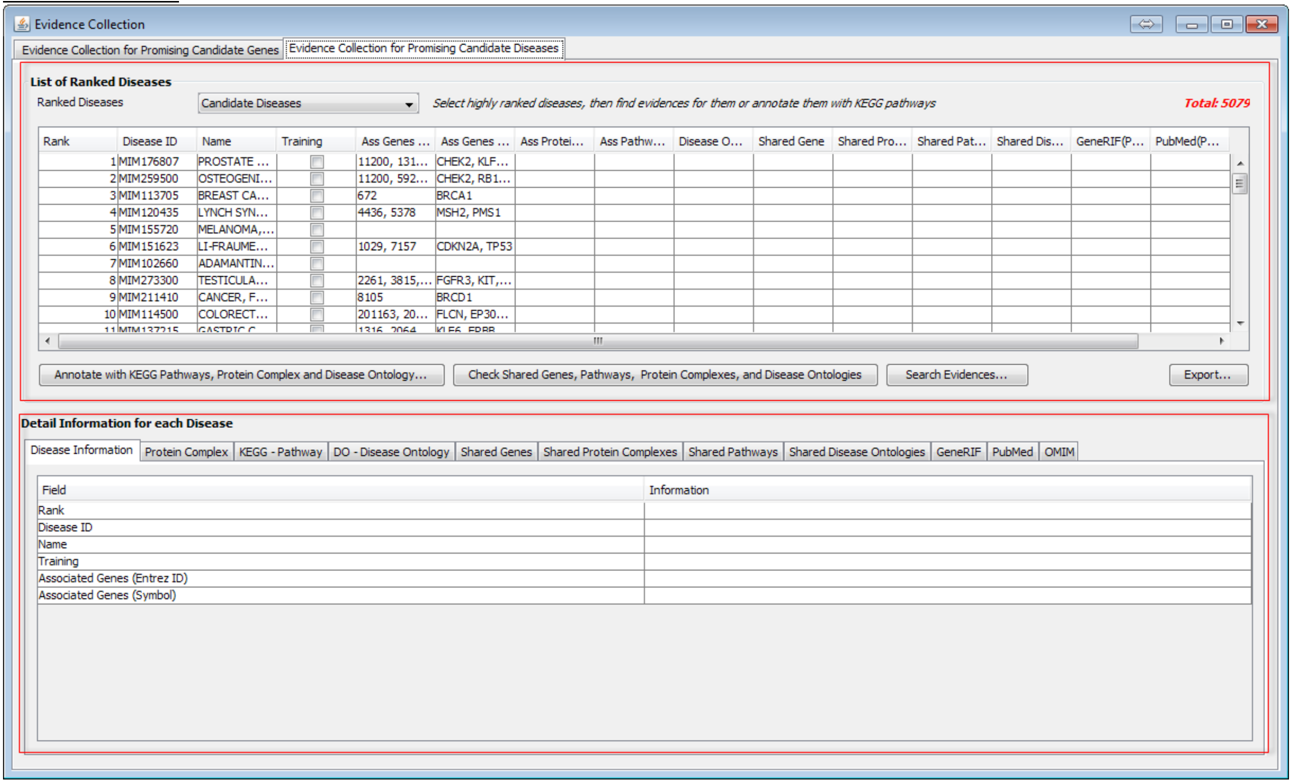

These two tabs are organized as follows:

- **Above panel** includes a table data of ranked genes/diseases and functional buttons
- **Below panel** includes tables of annotation and evidence data

Annotation and evidence collection for associations between highly ranked candidate genes and the disease of interest

We selected top 20 ranked candidate genes for evidence collection.

For annotation, this set can be easily annotated with pathways, protein complexes, disease ontology and gene ontology terms by click 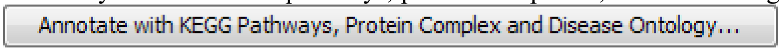

and 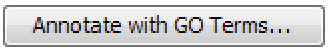

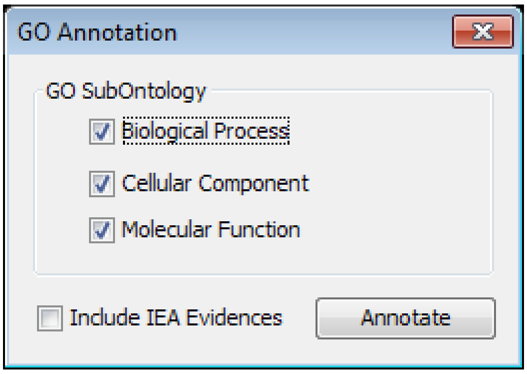

Select GO sub-ontology and whether or not IEA evidence is included. Then click 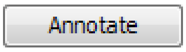

Note that:

- *GO Annotation data must be downloaded from ftp.ncbi.nlm.nih.gov/gene/DATA/gene2go.gz, then extracted and stored in Data folder in Cytoscape folder (e.g., C:\Program Files\Cytoscape_v2.8.3\Data).*

As a result, the top 20 ranked candidate genes are annotated with pathways, protein complexes, disease ontology and gene ontology

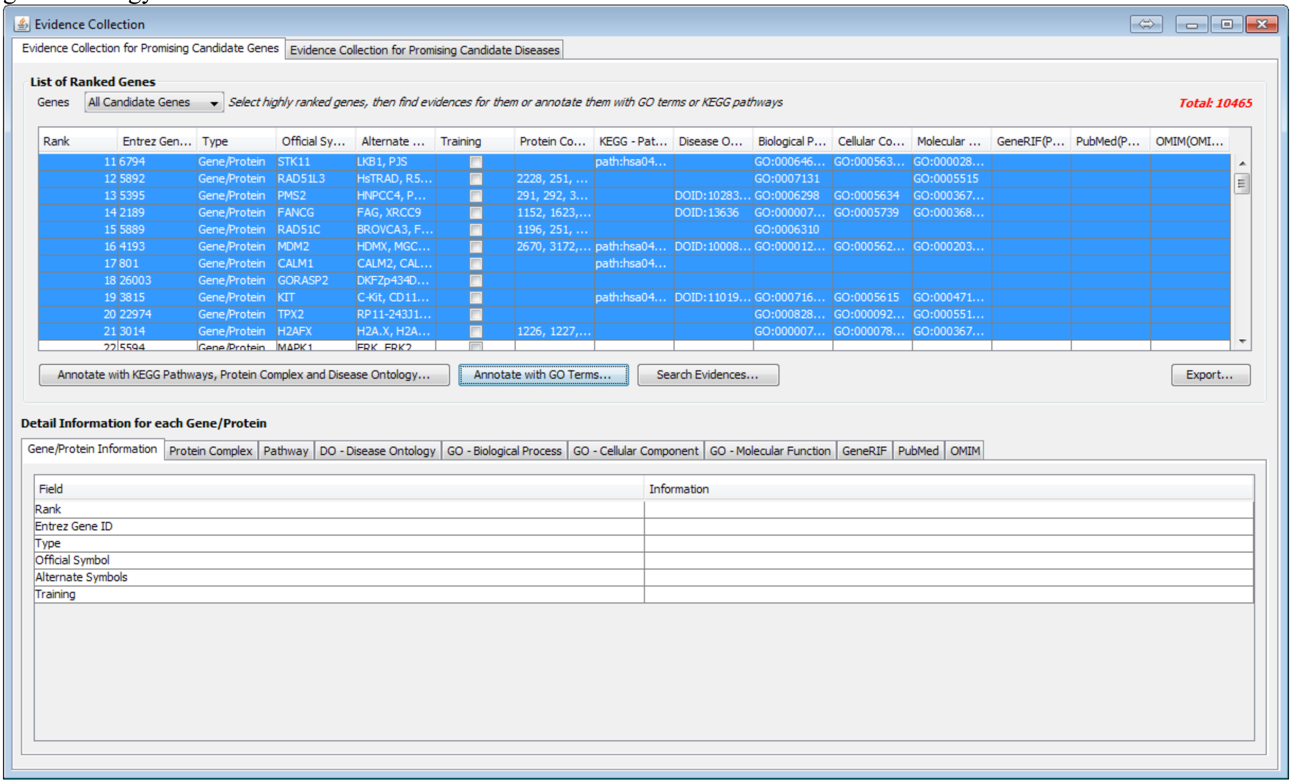

For association evidence search, promising associations between these selected candidate genes and the disease of interest can be collected from **GeneRIF** (Mitchell, et al., 2003; Osborne, et al., 2007), **PubMed** (Chang, et al., 2006) and **OMIM** (Amberger, et al., 2009; Hamosh, et al., 2005).

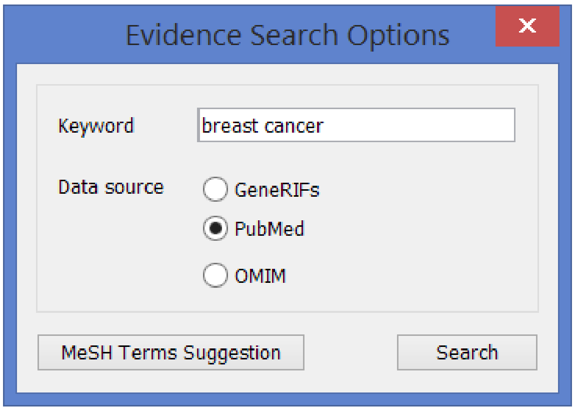

Enter a keyword for disease of interest or select a corresponding MeSH term by clicking [MeSH Terms Suggestion]. After that, select a data source among the three ones (i.e., GeneRIF, PubMed and OMIM), then click [Search]. Please wait while HPGEC is updating detail information about found PubMed and OMIM records.

Note that:

- *For GeneRIF, GeneRIF data must be downloaded from ftp://ftp.ncbi.nih.gov/gene/GeneRIF/generifs_basic.gz, then extracted and stored in Data folder in Cytoscape folder (e.g., C:\Program Files\Cytoscape_v2.8.3\Data).*
- *For PubMed and OMIM, HGPEC automatically search the association between the keyword and the disease of interest from PubMed and OMIM using NCBI web services.*

Here are the result after repeating the above procedure three times for the three data sources.

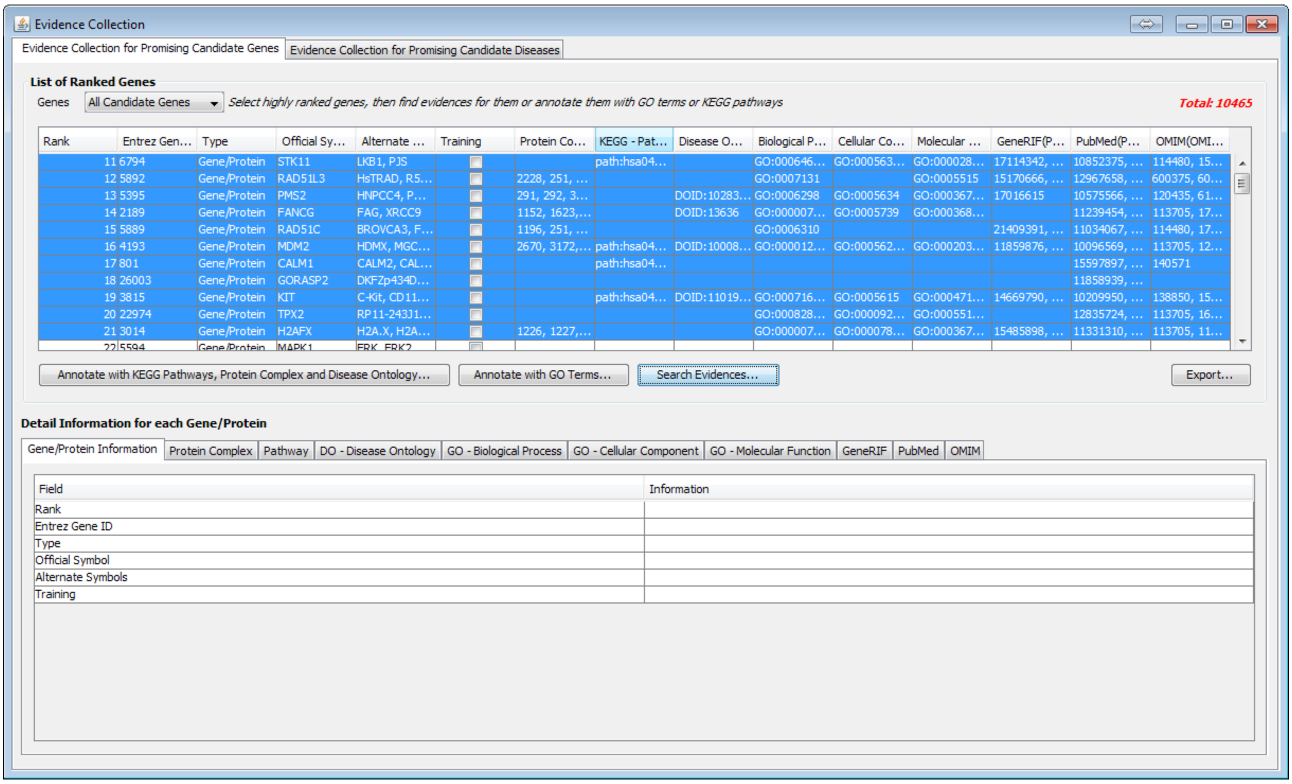

Detail information of annotations (pathway, protein complex, disease ontology and gene ontology) and evidences (PubMed articles, GeneRIF text and OMIM title) can be viewed in lower panels by selecting each gene. For example:

GO – Cellular component for gene RB1

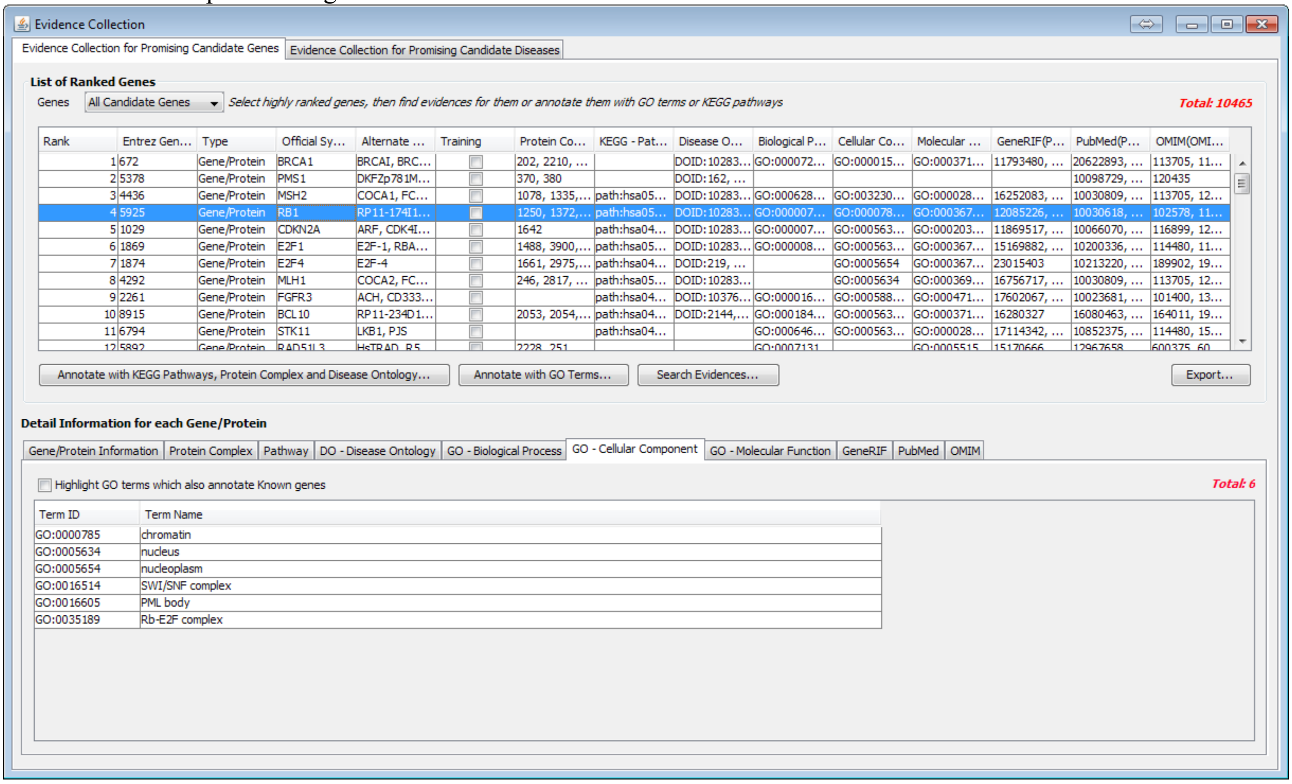

There are six GO cellular component terms annotating to RB1 gene

Evidences from GeneRIF data source for associations between gene RB1 and breast cancer

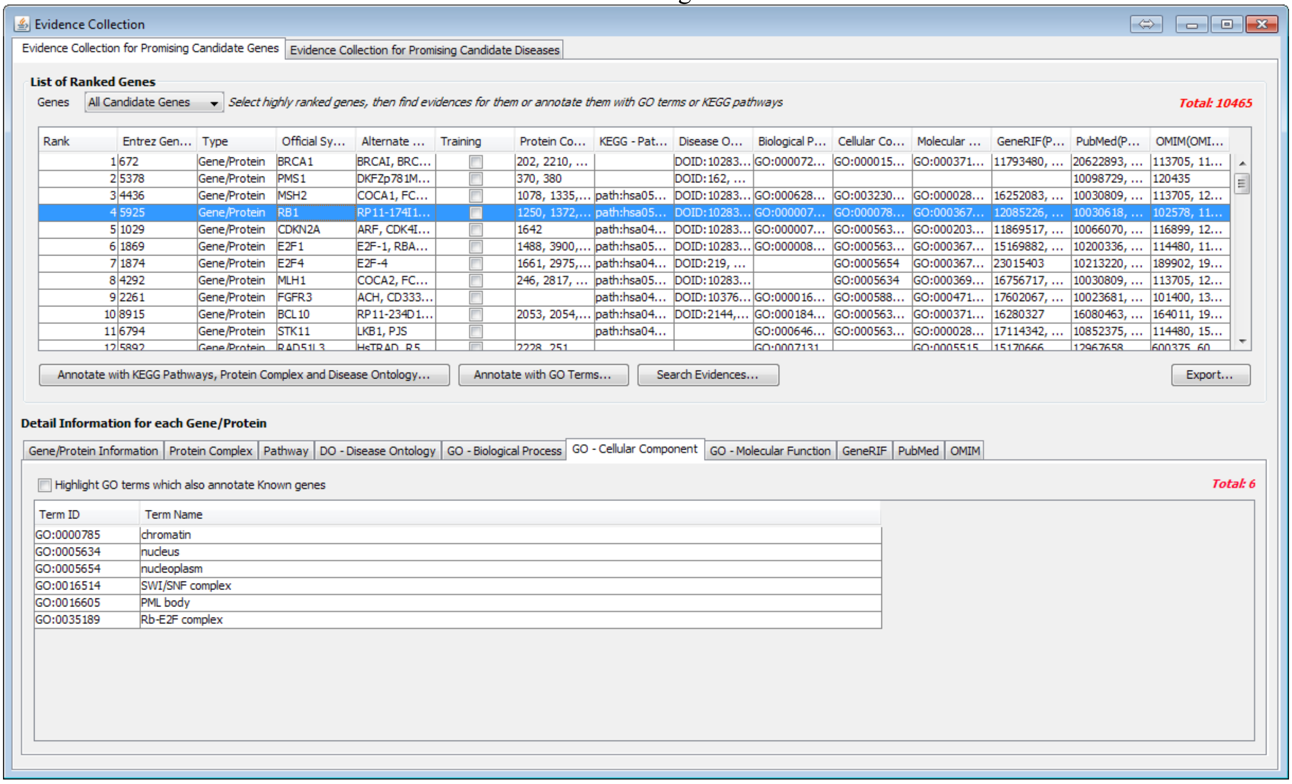

There are 20 PubMed articles whose abstracts contain information about associations between RB1 gene and breast cancer. GeneRIF text are sentences in the abstracts, which contains such the associations.

User can also export the selected candidate genes with their annotation and evidence information by clicking 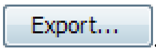

. See the exported file in Table S1 in Supplementary Materials

__Annotation and evidence collection for relevance between highly ranked candidate diseases and the disease of interest__

We selected top 20 ranked candidate diseases for annotation and evidence collection.

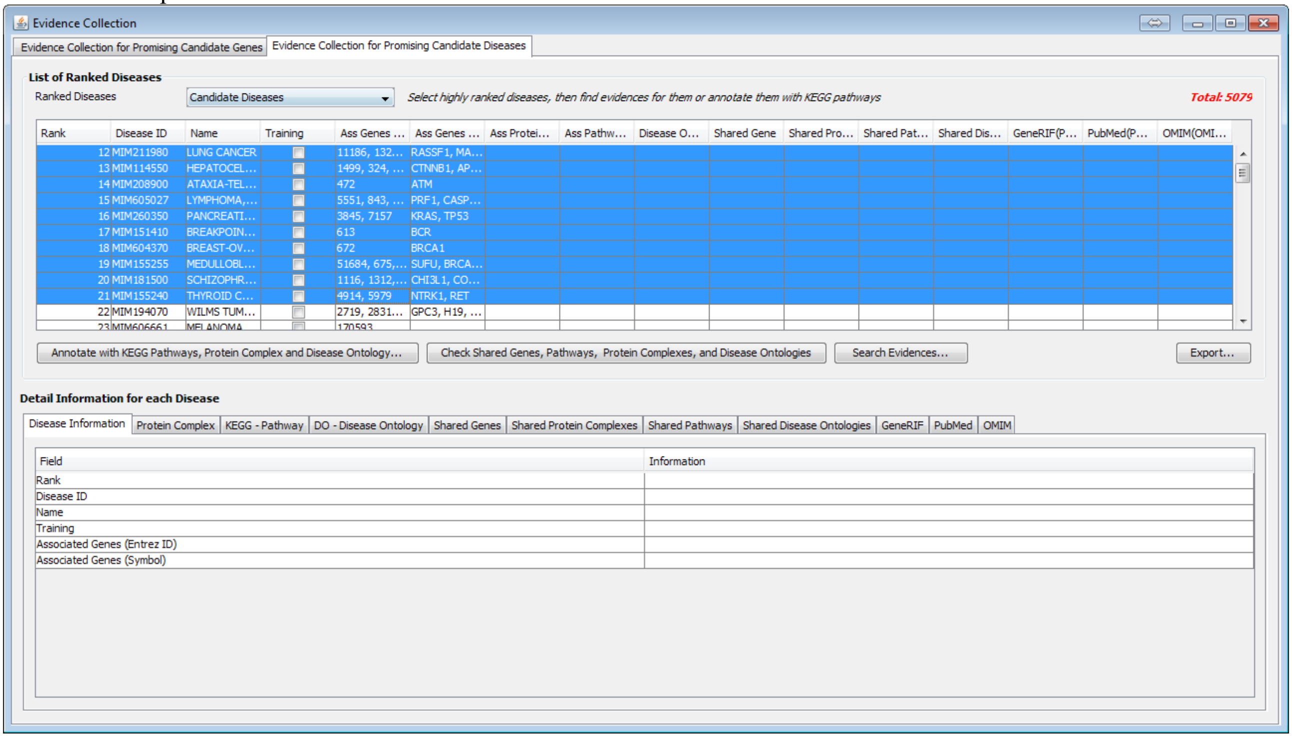

Similarly, this set can be easily annotated with pathways, protein complexes, disease ontologies and gene ontology (GO) terms by clicking Annotate with KEGG Pathways, Protein Complex and Disease Ontology...

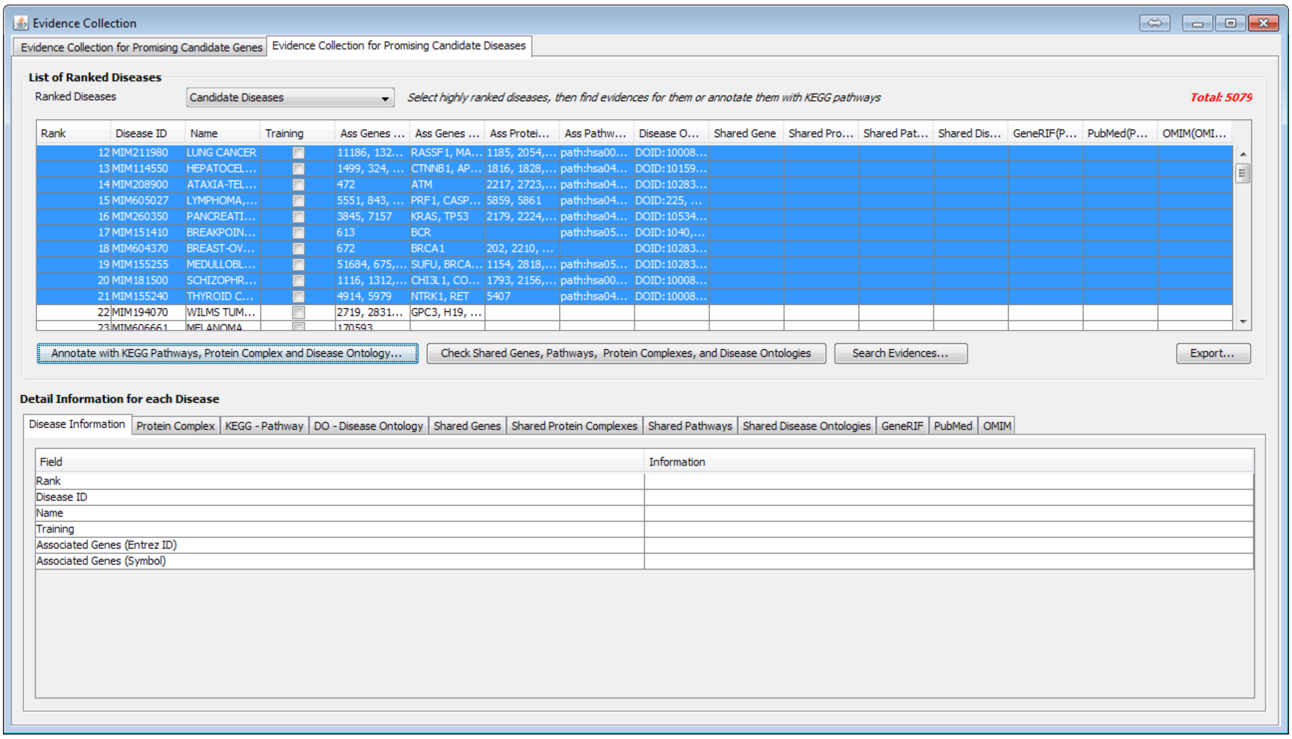

Note that, a number of studies have defined associations between diseases based on their shared associated genes (Goh, et al., 2007), shared protein complexes (Wang, et al., 2012), shared pathway (Li and Agarwal, 2009) and shared disease ontology (Li, et al., 2011). Therefore, in addition to annotate each disease in the top ranked candidate diseases with pathways, protein complexes and disease ontology terms, we provided a function to check whether or not these selected candidate diseases share genes, pathways, protein complexes and disease ontology terms with the disease of interest by clicking Check Shared Genes, Pathways, Protein Complexes, and Disease Ontologies

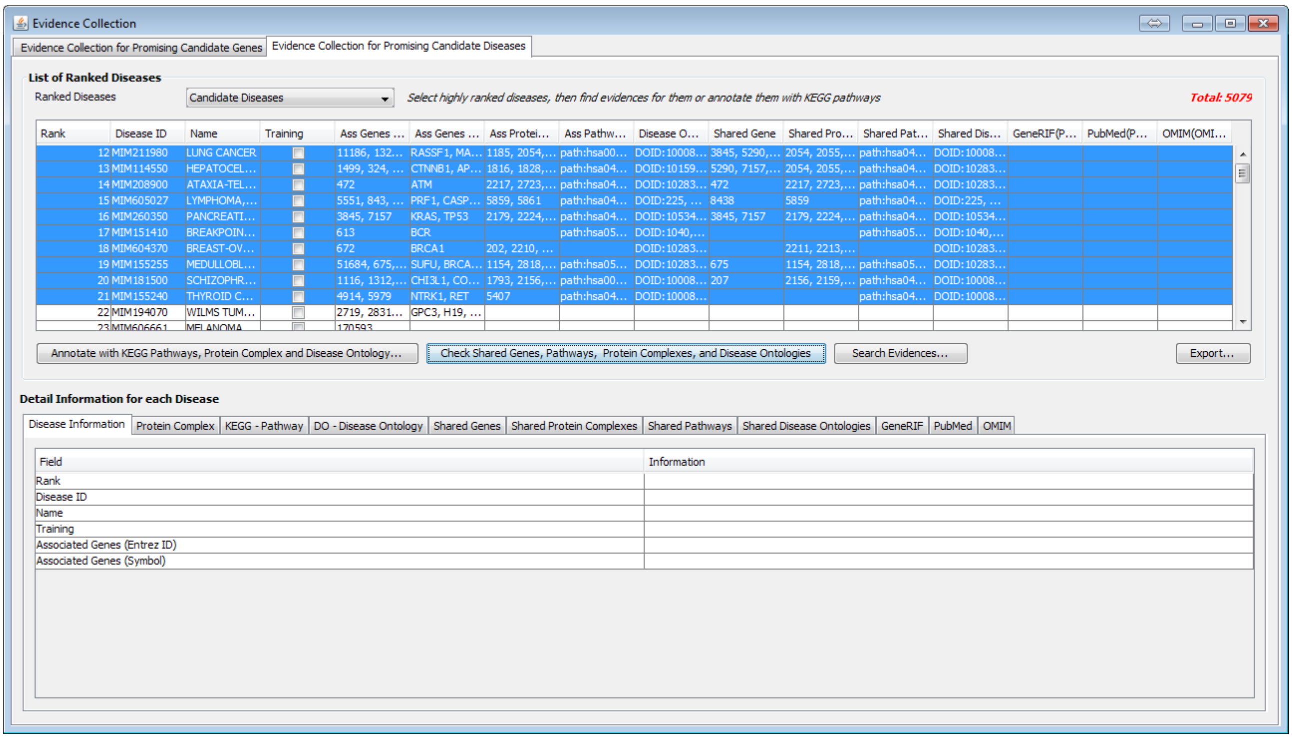

Further analyzing the above result, we found that

- Twelve of 20 selected candidate diseases (Ranks: 1, 2, 6, 10, 11, 12, 13, 14, 15, 16, 19 and 20) have at least one gene, pathway, protein complex and disease ontology term shared with the disease of interest.
- Five of 20 selected candidate diseases (Ranks: 3, 4, 8, 17 and 18) have at least one pathway, protein complex and disease ontology term shared with the disease of interest. However, they do not have any shared genes with the diseases of interest. This means that if we only based on the shared genes to associate these diseases with the disease of interest, we could not find any association. However, other biomedical data such as pathway, protein complex and disease ontology can provide evidences for their associations.
- The three remaining of 20 selected candidate diseases (Ranks: 5, 7 and 9) do not have any shared gene, shared pathway, shared protein complex and shared disease ontology term with the disease of interest. However, they still have high rankings, which indicates that they highly are associated with the disease of interest. Further investigating their topological relationships with the disease of interest, we found an interesting result that like others in the top 20 selected candidate diseases, these diseases directly connected to the disease of interest (breast cancer (OMIM ID: 114480)) in the phenotypic disease similarity network.

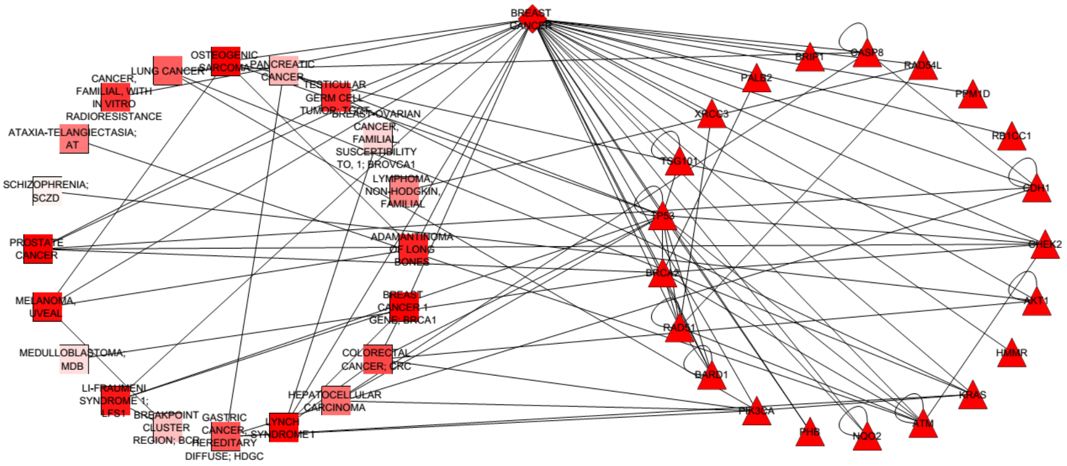

Similarly, information about associations between selected candidate diseases and the disease of interest can be collected from GeneRIF, PubMed and OMIM based on the associations between their known associated genes and the disease of interest.

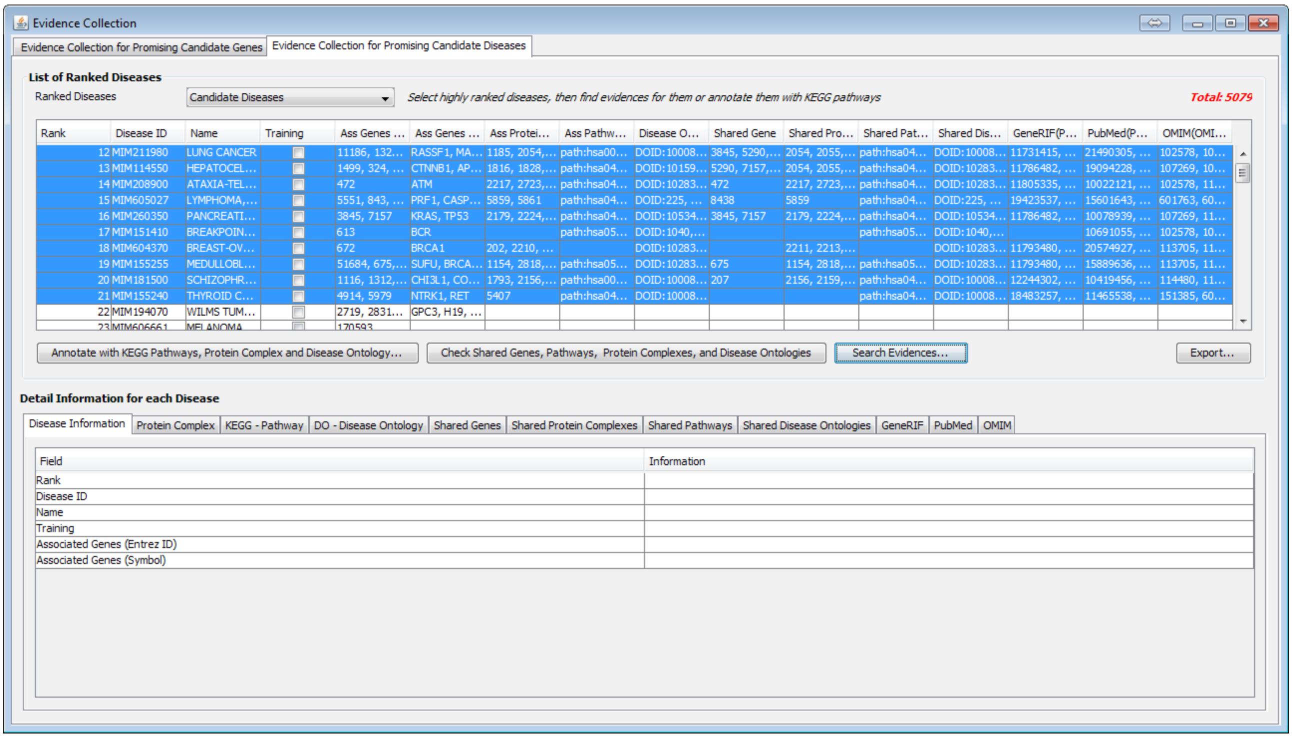

In a similar way, detail information of annotations (pathways, protein complex and disease ontology) and sharing (genes, pathways, protein complexes and disease ontology terms) can be viewed in lower panels by selecting each disease. For example, here is the result for Prostate cancer (OMIM ID: 176807)

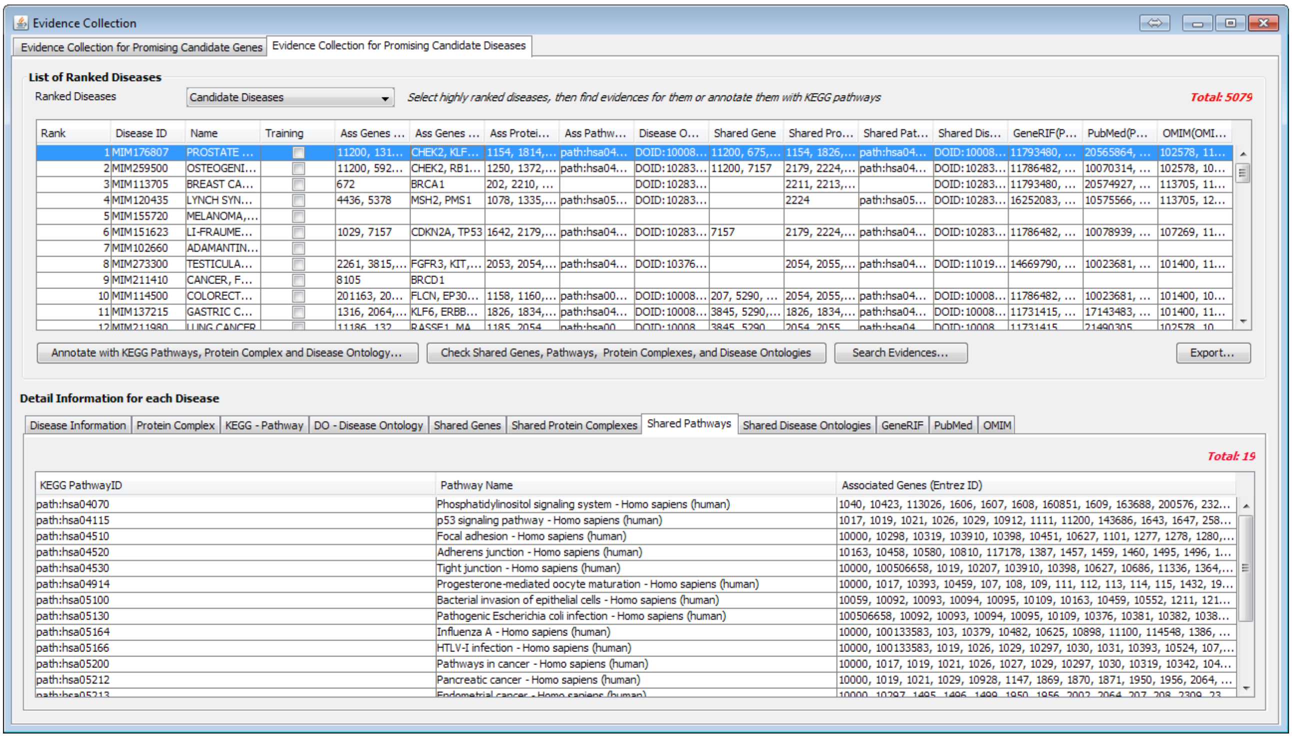

There are 19 common pathways between Prostate cancer (OMIM ID: 176807) and Breast cancer (OMIM ID: 114480)

Similarly, user can also export the selected candidate diseases with their annotation and evidence information by clicking 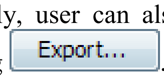. See the exported file in Table S2 in Supplementary Materials.

IV. Reference

Amberger, J., *et al.* (2009) McKusick's Online Mendelian Inheritance in Man (OMIM®), *Nucleic Acids Research*, **37**, D793-D796.

Chang, A.A., Heskett, K.M. and Davidson, T.M. (2006) Searching the Literature Using Medical Subject Headings versus Text Word with PubMed, *The Laryngoscope*, **116**, 336-340.

Goh, K.-I., *et al.* (2007) The human disease network, *Proceedings of the National Academy of Sciences*, **104**, 8685-8690.

Hamosh, A., *et al.* (2005) Online Mendelian Inheritance in Man (OMIM), a knowledgebase of human genes and genetic disorders, *Nucleic Acids Research*, **33**, D514-517.

Li, J., *et al.* (2011) DOSim: An R package for similarity between diseases based on Disease Ontology, *BMC Bioinformatics*, **12**, 266.

Li, Y. and Agarwal, P. (2009) A Pathway-Based View of Human Diseases and Disease Relationships, *PLoS ONE*, **4**, e4346.

Li, Y. and Patra, J.C. (2010) Genome-wide inferring gene-phenotype relationship by walking on the heterogeneous network, *Bioinformatics*, **26**, 1219-1224.

Mitchell, J.A., *et al.* (2003) Gene Indexing: Characterization and Analysis of NLM's GeneRIFs. In, *Proceedings of AMIA 2003 Symposium*. American Medical Informatics Association.

Osborne, J., *et al.* (2007) GeneRIF is a more comprehensive, current and computationally tractable source of gene-disease relationships than OMIM, *Bioinformatics Core, Northwestern University*, **Technical Report**.

Piñero, J., *et al.* (2017) DisGeNET: a comprehensive platform integrating information on human disease-associated genes and variants, *Nucleic Acids Research*, **45**, D833-D839.

van Driel, M.A., *et al.* (2006) A text-mining analysis of the human phenome, *Eur J Hum Genet*, **14**, 535-542.

Wang, Q., *et al.* (2012) Community of protein complexes impacts disease association, *Eur J Hum Genet*, **20**, 1162-1167.
